# Restoring mitochondrial function promotes hematopoietic reconstitution from cord blood following cryopreservation-related functional decline

**DOI:** 10.1172/JCI183607

**Published:** 2025-03-04

**Authors:** Yaojin Huang, Xiaowei Xie, Mengyao Liu, Yawen Zhang, Junye Yang, Wenling Yang, Yu Hu, Saibing Qi, Yahui Feng, Guojun Liu, Shihong Lu, Xuemei Peng, Jinhui Ye, Shihui Ma, Jiali Sun, Lu Wang, Linping Hu, Lin Wang, Xiaofan Zhu, Hui Cheng, Zimin Sun, Junren Chen, Fang Dong, Yingchi Zhang, Tao Cheng

**Affiliations:** 1 State Key Laboratory of Experimental Hematology, National Clinical Research Center for Blood Diseases, Haihe Laboratory of the Cell Ecosystem, Institute of Hematology and Blood Diseases Hospital, Chinese Academy of Medical Sciences and Peking Union Medical College, Tianjin, China.; 2 Tianjin Institutes of Health Science, Tianjin, China.; 3 Department of Hematology, Jiangsu Province Hospital, The First Affiliated Hospital with Nanjing Medical University, Nanjing, China.; 4 Tianjin Cord Blood Stem Cell Bank, Tianjin, China.; 5 Union Stem Cell Genetic Engineering Co., Ltd., Tianjin, China.; 6 Shandong Qilu Stem Cell Engineering Co., Ltd., Jinan, China.; 7 Institute of Basic Medical Sciences, Chinese Academy of Medical Sciences and Peking Union Medical College, Beijing, China.; 8 Department of Hematology, The First Affiliated Hospital of University of Science and Technology of China, Hefei, China.; 9 Blood and Cell Therapy Institute, Division of Life Sciences and Medicine, Anhui Provincial Key Laboratory of Blood Research and Applications, University of Science and Technology of China, Hefei, China.

**Keywords:** Hematology, Stem cells, Hematopoietic stem cells

## Abstract

Umbilical cord blood (UCB) plays substantial roles in hematopoietic stem cell (HSC) transplantation and regenerative medicine. UCB is usually cryopreserved for years before use. It remains unclear whether and how cryopreservation affects UCB function. We constructed a single-cell transcriptomics profile of CD34^+^ hematopoietic stem and progenitor cells (HSPCs) and mononuclear cells (MNCs) from fresh and cryopreserved UCB stored for 1, 5, 10, and 19 years. Compared with fresh UCB, cryopreserved HSCs and multipotent progenitors (MPPs) exhibited more active cell-cycle and lower expression levels of HSC and multipotent progenitor signature genes. Hematopoietic reconstitution of cryopreserved HSPCs gradually decreased during the first 5 years but stabilized thereafter, aligning with the negative correlation between clinical neutrophil engraftment and cryopreservation duration of UCB. Cryopreserved HSPCs also showed reduced megakaryocyte generation. In contrast, cryopreserved NK cells and T cells maintained a capacity for cytokine production and cytotoxicity comparable to that of fresh cells. Mechanistically, cryopreserved HSPCs exhibited elevated ROS, reduced ATP synthesis, and abnormal mitochondrial distribution, which collectively led to attenuated hematopoietic reconstitution. These effects could be ameliorated by sulforaphane (SF). Together, we elucidate the negative effect of cryopreservation on UCB HSPCs and identify SF as a mitigation strategy, broadening the temporal window and scope for clinical applications of cryopreserved UCB.

## Abstract 2

Unlike UCB T/NK cells, cryopreservation affected the reconstitution of UCB HSPCs, whose mitochondrial dysfunction could be mitigated by sulforaphane.

## Graphical abstract

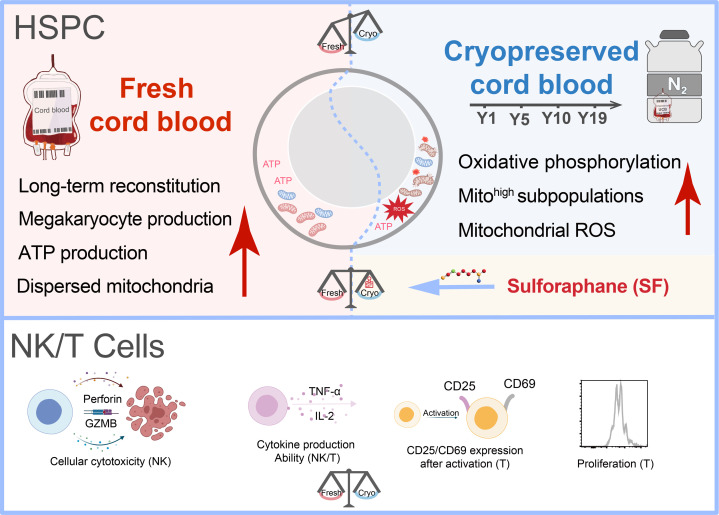

## Introduction

Umbilical cord blood (UCB) is characterized by abundant hematopoietic stem and progenitor cells (HSPCs), naive lymphocytes, and divergent non-blood cell components, including mesenchymal stem cells, endothelial progenitor cells, and very small embryo-like stem cells (1–4). In the beginning, UCB transplantation (UCBT) was conducted for curing blood diseases because of its abundance of HSPCs with a less stringent HLA match, less severe chronic graft-versus-host disease (GVHD), and more a potent graft-versus-leukemia effect in patients with acute myeloid leukemia than was seen with allogeneic stem cell transplantation (5–10). Recently, mature blood cells in UCB have also provided a promising resource for CAR-T cell and CAR-NK cell therapies (11–14). However, only approximately 40,000 UCBTs have been clinically performed to tackle hematologic, immunologic, and neurologic diseases (7, 15, 16), despite there being more than 5 million UCB resources stored worldwide today. One of the reasons for this lack of application is that, in clinical practice, doctors prefer UCB that has been cryopreserved for a shorter duration. Whether and how cryopreservation over time influence the function of HSPCs and other mature blood cells still need further investigation.

UCB is usually cryopreserved in liquid nitrogen until it is thawed for use, thus the recovery efficiency of cryopreserved UCB is crucial for UCB banking and clinical transplantation. Hematopoietic progenitor cells (HPCs) from UCB cryopreserved for 5, 10, or 15 years have reportedly been efficiently recovered (17–19). Even HSPCs isolated from UCB cryopreserved for more than 20 years still showed sustained repopulating capabilities in the mouse model (20, 21). Nevertheless, these studies mainly focused on the recovered cell numbers and reconstruction ability of cryopreserved UCB-derived HSPCs, while the parallel comparison designed to investigate the dynamic influence of cryopreservation on UCB stored for different durations was lacking. In addition, the alteration of mature blood cells in UCB after cryopreservation, such as NK and T cells, which have become potential resources for immune therapy in recent years, was poorly understood.

Here, we examined the effect of cryopreservation with various storage periods on different components of UCB and the underlying mechanisms through side-by-side comparisons with fresh counterparts. First, we established the single-cell transcriptomics landscape of hematopoietic cells derived from fresh and cryopreserved UCB stored for 1, 5, 10, and 19 years. Then, the functional alteration of HSPCs and mature T and NK cells was assessed to validate the transcriptional changes, followed by investigation of the underlying molecular mechanism. More important, we validated a strategy to mitigate the negative effects of cryopreservation on HSPCs. In short, our work provides insights into the selection of cryopreserved UCB used in the clinic and offers an effective strategy to improve the quality of cryopreserved UCB.

## Results

### Single-cell transcriptomics landscape of CD34^+^ HSPCs and mononuclear cells from fresh and cryopreserved UCB.

**Figure 1:**
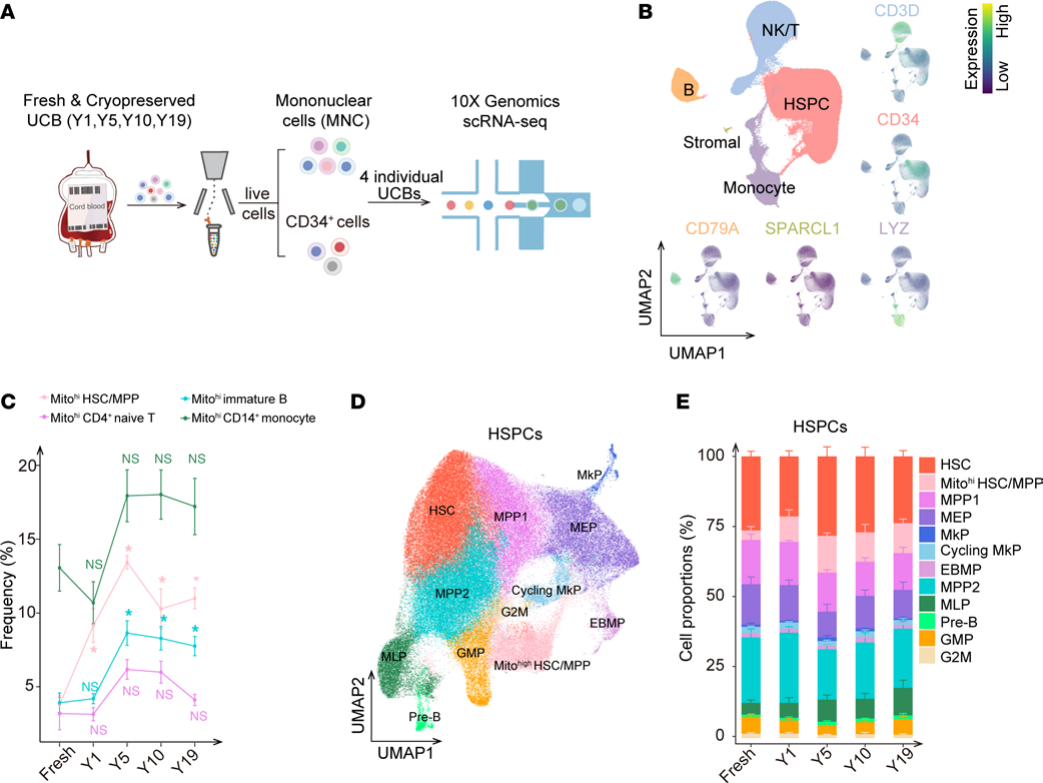
Characterization of subclusters with high expression levels of mitochondrial genes. (**A**) Schematic diagram showing the sequencing design. Live CD34^+^ cells and MNCs from fresh and cryopreserved UCB stored for 1 (Y1), 5 years (Y5), 10 years (Y10), and 19 years (Y19) (*n* = 4 biological replicates for each storage period) were sorted to perform single-cell RNA-Seq (scRNA-Seq) using the 10x Genomics platform. (**B**) UMAP plot displaying the distributions of 5 cell populations from all UCB samples. The surrounding UMAP plots display the expression levels of signature genes used to identify each cell population: HSPCs (characterized by CD34 expression), T and NK cells (characterized by CD3D and KLRD1 expression), B cells (characterized by CD79A expression), monocytes (characterized by lysozyme [LYZ] expression), and stromal cells (characterized by secreted protein acidic and rich in cysteine-like 1 [SPARCL1] expression). (**C**) The proportion changes of the Mito^hi^ subpopulations over time. The proportions of Mito^hi^ cell clusters at 1 year, 5 years, 10 years, and 19 years were compared with those of fresh samples using the Wilcoxon test (*P ≤ 0.05). (**D**) UMAP plot displaying the distributions of 12 cell clusters of HSPCs. Colors indicate cell clusters determined by unsupervised Leiden clustering. (**E**) Cellular compositions of 12 cell clusters in HSPCs derived from fresh UCB and UCB cryopreserved for 1, 5, 10, or 19 years (*n* = 4 replicates; data indicate the mean ± SD). The cellular abundances of different subclusters at 1 year, 5 years, 10 years, and 19 years were respectively compared with that of fresh UCB using the χ^2^ test (see the Supporting Data Values file).

We first confirmed the comparable cell viability among fresh and cryopreserved UCB samples (Supplemental Figure 1A; supplemental material available online with this article; https://doi.org/10.1172/JCI183607DS1). To comprehensively dissect the alterations occurring to heterogenous hematopoietic cells after cryopreservation, we constructed the single-cell transcriptomics profile of CD34^+^ HSPCs and mononuclear cells (MNCs) derived from fresh UCB and UCB cryopreserved for 1, 5, 10, and 19 years (4 individual donors were used for each cryopreservation duration) (Figure 1A and Supplemental Figure 1, B and C), with well batch correction (Supplemental Figure 1, D–G). Projection onto the Atlas of Blood Cells (ABC) (22) in combination with uniform manifold approximation and projection (UMAP) visualization for canonical signature genes contributed to the accurate annotation of the UCB hematopoietic cell identities. In total, 143,385 HSPCs, 79,938 T/NK cells, 22,938 B cells, and 36,844 monocytes were harvested for further subclustering, as well as a few stromal cells (Figure 1B and Supplemental Data File 1). Within each compartment, we identified cell subpopulations with high expression levels of mitochondrial genes (namely Mito^hi^ hematopoietic stem cell/multipotent progenitor [HSC/MPP], Mito^hi^ CD4^+^ naive T cell, Mito^hi^ immature B cell, and Mito^hi^CD14^+^ monocyte populations) and an increased tendency of cell proportions after cryopreservation (Figure 1, C and D, and Supplemental Figure 2, A–G).

For the HSPC compartment, HSC/MPP clusters were characterized by expression of arginine vasopressin (AVP); corticotropin releasing hormone binding protein (CRHBP); and myeloid/lymphoid or mixed-lineage leukemia, translocated to 3 (MLLT3); and the megakaryocyte-erythroid progenitor (MEP) cluster was identified by its high expression of GATA binding protein 1 (GATA1), catenin beta like 1 (CTNNBL1), and uroporphyrinogen decarboxylase (UROD). Integrin subunit alpha 2b (ITGA2B)/glycoprotein IX platelet (GP9) and histidine decarboxylase (HDC)/tryptase beta 2 (TPSB2) were used to identify megakaryocyte progenitors (MkPs) and eosinophil/basophil/mast cell progenitors (EBMPs), respectively. Myeloperoxidase (MPO)/lysozyme (LYZ) and marginal zone B and B1 cell-specific protein (MZB1)/brain and acute leukemia, cytoplasmic (BAALC) were respectively activated in the granulocyte-macrophage progenitor (GMP) and multilymphoid progenitor (MLP) clusters (Figure 1D and Supplemental Figure 2A). Compared with fresh UCB-derived HSPCs, the cellular abundance of Mito^hi^ HSCs/MPPs and MLPs was increased, whereas that of MEPs was slightly decreased during cryopreservation (Figure 1E and Supporting Data Values). Similarly, Mito^hi^ CD4^+^ naive T cells, Mito^hi^ immature B cells, and Mito^hi^ CD14^+^ monocytes were enriched after cryopreservation, suggestive of the universal response of UCB to cryopreservation (Figure 1C and Supplemental Figure 2, H–J). Although Mito^hi^ HSC/MPPs and Mito^hi^ CD14^+^ monocytes accounted for a large proportion of HSPCs and monocytes (Figure 1C), Mito^hi^ HSC/MPPs exhibited more significant quantitative changes and higher mitochondrial gene expression (Figure 1E and Supplemental Figure 2, A, G, and J), implying that HSPCs were more susceptible to cryopreservation than were mature blood cells. To remove the bias caused by the different UCB sources, CD34^+^ HSPCs and MNCs derived from another UCB bank in China, Shandong Qilu Stem Cell Engineering Co., Ltd., were collected and integrated together (4 individual donors were used for UCB cryopreserved for 10 years). The Mito^hi^ HSC/MPP subpopulation was still present at an enhanced cellular abundance after 10 years of cryopreservation (Supplemental Figure 2K). In addition, the number of apoptotic cells in the fresh UCB was comparable to that in the cryopreserved samples (Supplemental Figure 2L), implying that these Mito^hi^ subpopulations were not caused by apoptosis. Collectively, we characterized the heterogeneity and temporal dynamics of HSPCs and MNCs in cryopreserved UCB and identified a new subpopulation with high mitochondrial gene expression within each hematopoietic cell compartment from UCB. We discovered that the abundance of Mito^hi^ HSCs/MPPs was the most markedly increased cell population during cryopreservation.

### Substantial transcriptomic variations occur during the first year of cryopreservation.

**Figure 2:**
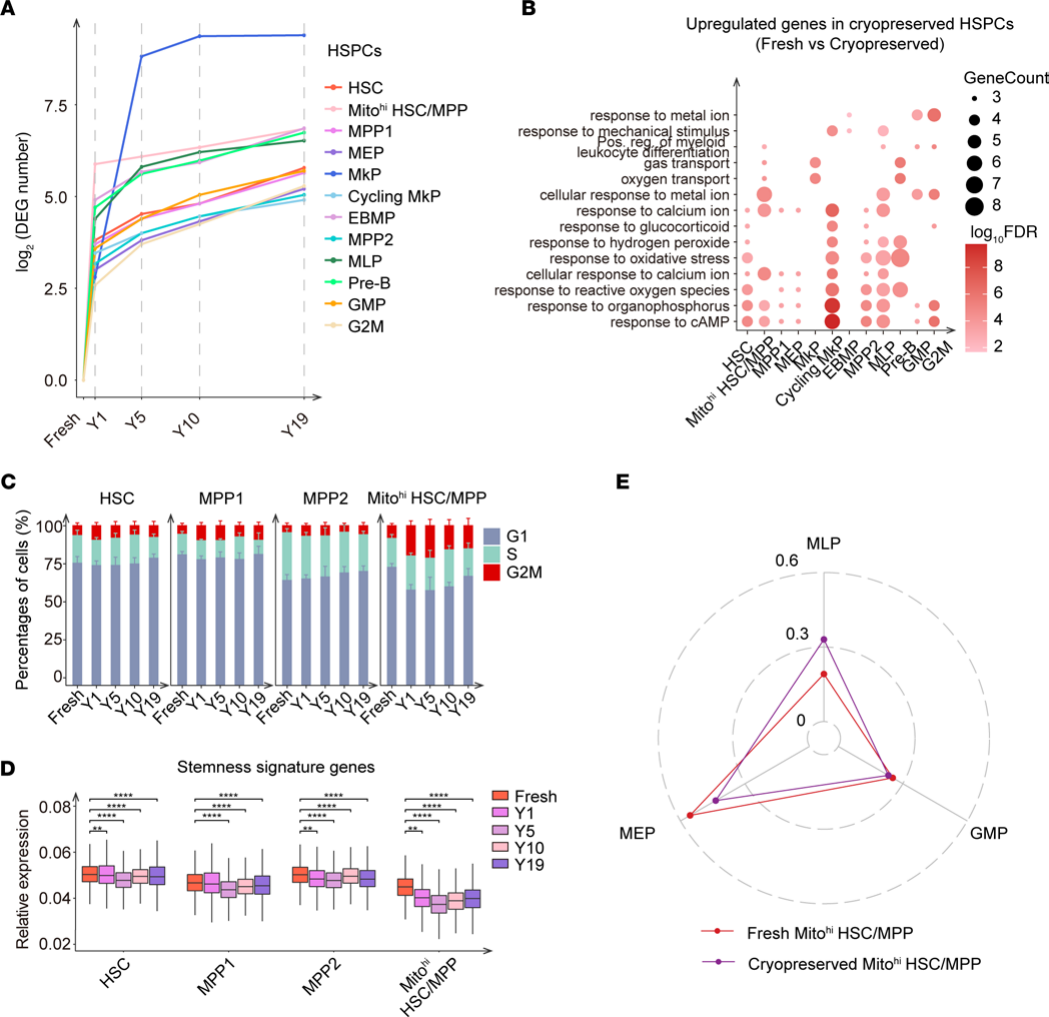
Substantial transcriptomic variations occur in HSPCs after cryopreservation. (**A**) The number of DEGs in HSPCs between 2 consecutive periods. (**B**) Bubble plot showing the enriched GO terms of upregulated genes in HSPCs from cryopreserved UCB (cryopreserved UCB vs. fresh UCB; UCB from different cryopreservation years was analyzed as a whole). Pos. reg., positive regulation. (**C**) Percentages of HSCs, MPP1s, MPP2s, and Mito^hi^ HSCs/MPPs in different cell-cycle phases for fresh and cryopreserved UCB stored for 1, 5, 10, and 19 years. For each cluster, the proportions of cells in the G_2_M phase at 1 year, 5 years, 10 years, and 19 years were respectively compared with that of fresh UCB using the χ^2^ test; data indicate the mean ± SD (see the Supporting Data Values file). (**D**) Relative expression of stemness signature genes in HSCs, MPP1s, MPP2s, and Mito^hi^ HSCs/MPPs from fresh and cryopreserved UCB stored for 1, 5, 10, and 19 years. **P ≤ 0.01 and ****P ≤ 0.0001, by Wilcoxon test. The stemness signature genes used for the analysis are listed in Supplemental Data File 2. (**E**) Potential of Mito^hi^ HSC/MPP differentiation toward MLP, GMP, and MEP lineages for fresh and cryopreserved UCB. UCB samples from different cryopreservation years were analyzed as a whole.

To evaluate the transcriptomic variations in each cell cluster, we determined differential gene expression between each cryopreservation duration with the other cryopreservation durations. We detected a higher median number of differentially expressed genes (DEGs) in Mito^hi^ subpopulations during cryopreservation (Supplemental Figure 3A), and the transcriptomic variations between fresh and 1-year cryopreserved UCB had greater changes than those between UCB stored for longer cryopreservation periods (Figure 2A and Supplemental Figure 3B). Considering the susceptibility of HSPCs to cryopreservation conditions and the pivotal role of HSPCs in long-term hematopoietic reconstruction, we conducted gene ontology (GO) analysis to ascertain the transcriptomic variations that emerged in HSPCs during their cryopreservation. We observed that upregulated genes in cryopreserved HSPCs showed enrichment in terms related to oxidative metabolism, whereas genes responsible for translation enrichment in HSC clusters (23) were transcriptionally activated in fresh UCB–derived HSPCs (Figure 2B and Supplemental Figure 3C).

Next, we detected higher percentages of Mito^hi^ HSCs/MPPs in the G_2_/M phase after cryopreservation compared with the cells from the fresh UCB group (Figure 2C and Supporting Data Values). Importantly, we found that the expression levels of HSC/MPP signature genes were markedly reduced after short-term cryopreservation (Figure 2D), which might be attributable to the more active cycling. According to the cell fate bias inferred by FateID (24), the HSC/MPP clusters, especially Mito^hi^ HSC/MPPs, showed a lower capacity for differentiation into Mks after cryopreservation (Figure 2E and Supplemental Figure 3D), mirroring the notable transcriptomic variation in MkPs during cryopreservation (Figure 2A). Furthermore, HSC/MPP clusters from cryopreserved UCB collected from Shandong Qilu Stem Cell Engineering Co. displayed the same changes as those in cells from UCB from the Tianjin Cord Blood Stem Cell Bank (Supplemental Figure 3, E–I). Together, the evidence showed that drastic transcriptomic variations occurred in the HSC/MPP clusters during the first year of cryopreservation, mainly manifesting as a more active cell cycle, lower expression levels of HSC/MPP signature genes, and a decreased differentiation bias toward Mks.

### Gradual attenuation of long-term reconstitution and self-renewal ability of HSPCs within the first 5 years of UCB cryopreservation.

**Figure 3:**
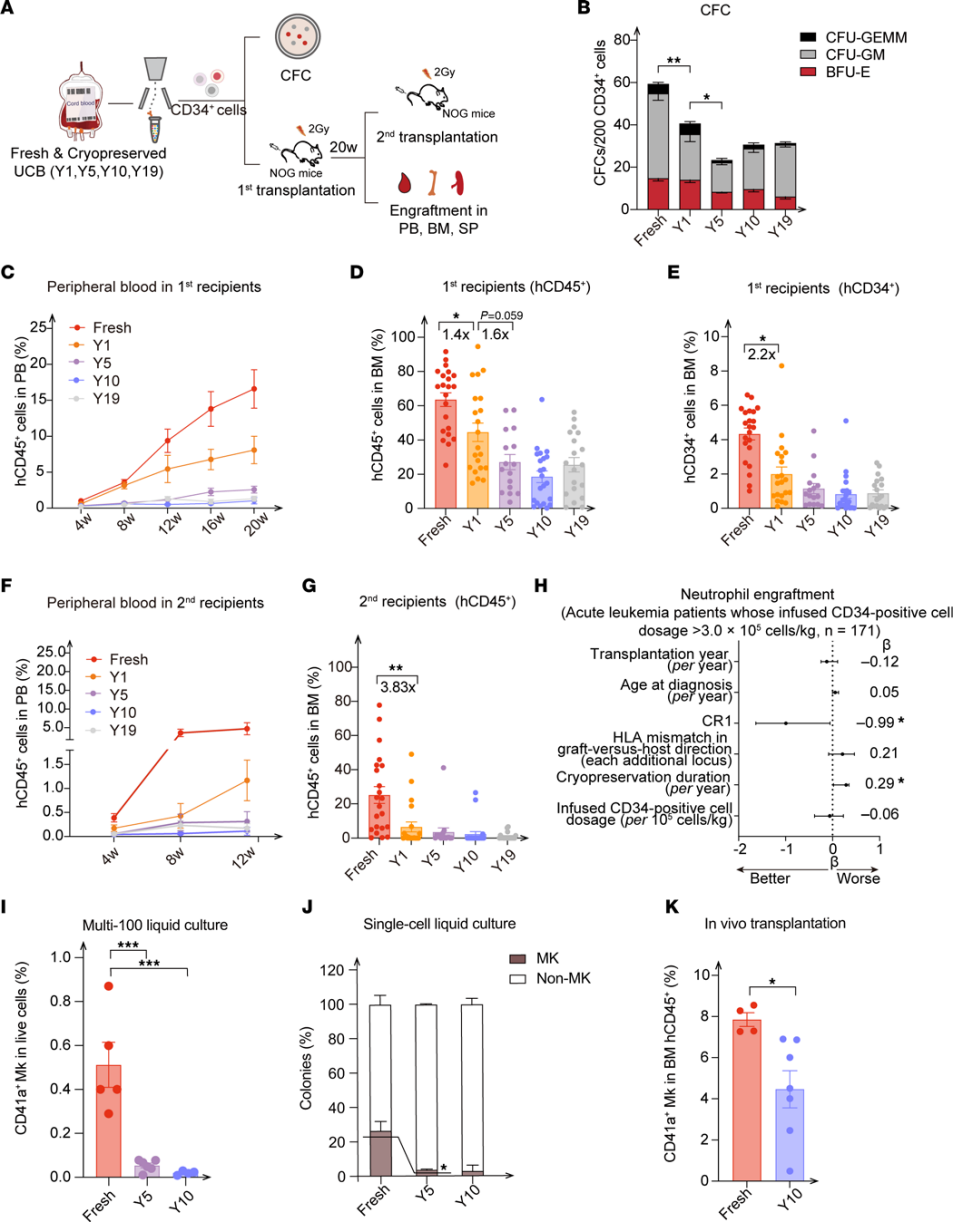
Decreased hematopoietic reconstitution and Mk production of HSPCs during the first 5 years of cryopreservation. (**A**) Experimental design for the functional studies of HSPCs from fresh and cryopreserved UCB. Live CD34^+^ cells were sorted by FACS. For in vitro assays, CFU assays were performed on CD34^+^ cells from individual UCB samples. For in vivo analysis, 4–6 pooled UCB samples (same storage duration) were transplanted into NOG mice. For the primary transplantation, 36,000 CD34^+^ cells per mouse (*n* = 15–24 recipient mice/group) were injected via the tail vein. For the secondary transplantation, 1 × 10^7^ cells from each primary recipient were transplanted into second recipients. CFC, colony-forming cell; 20W, 20 weeks. (**B**) CFCs per 200 CD34^+^ cells from fresh and cryopreserved UCB stored for different durations (years) (fresh: *n* = 8; 1 year, 5 years: *n* = 5; 10 years: *n* = 12; 19 years: *n* = 6). (**C**–**G**) Percentages of hCD45^+^ or hCD34^+^ cells engrafted in the PB or BM of primary (**C**–**E**) and secondary (**F** and **G**) recipient mice. (**H**) Cryopreservation duration predicted neutrophil engraftment. The multivariate model was fitted using the quantile regression method. (**I**) Lineage output of Mks (CD41a^+^) from bulk (*n* = 100 cells) CD34^+^ cells from fresh UCB and cryopreserved UCB (*n* = 4–5 replicates, 2 independent experiments). (**J**) Percentage of Mk versus non-Mk colonies in fresh and cryopreserved UCB (fresh: *n* = 40 colonies; 5 years: *n* = 52 colonies; 20 years: *n* = 34 colonies). (**K**) Percentage of human CD41a^+^ Mk cells engrafted in the BM of recipients (NCG-X mice). *n* = 4 pooled UCB samples; *n* = 4–7 recipient mice per group. For the transplantation assay, UCB-derived CD34^+^ cells (40,000 cells/mouse) were injected via the tail vein. (**C**–**G**) *n* = 4–6 pooled UCB samples; *n* = 15–24 recipient mice per group. Data indicate the mean ± SEM. *P ≤ 0.05, **P ≤ 0.01, and ***P ≤ 0.001, by 1-way ANOVA or Kruskal-Wallis test with post hoc Tukey’s test applied for multiple comparisons (**C**–**G** and **I**) and 2-tailed *t* test (**J** and **K**).

The active cell cycle and diminished expression levels of HSC/MPP signature genes prompted us to detect cell function in cryopreserved UCB. CD34^+^ HSPCs from fresh and cryopreserved UCB for different durations were individually sorted, and then subjected to an independent CFU assay (Figure 3A). In contrast to HSPCs from the fresh UCB group, cryopreserved HSPCs exhibited a gradual decline in colony-forming ability during the first 5 years of storage but showed no significant decrease after 5 years (Figure 3B and Supplemental Figure 4A). Because of the low variation of different biological replicates in the in vitro assay and the limited number of HSPCs in a single unit of UCB (Supplemental Figure 4A), we used pooled UCBs that had the same storage duration for the in vivo serial transplantation assays (Figure 3A). The long-term engraftment capacity of cryopreserved HSPCs gradually decreased within the first 5 years in the peripheral blood (PB), bone marrow (BM), and spleen (SP) of recipients, while maintaining similar engraftment capacities thereafter (Figure 3, C–E, and Supplemental Figure 4, B and C). Of note, there were consecutive 1.4-fold and 1.6-fold decreases in total engraftment in fresh UCB HSPCs compared with HSPCs from UCB cryopreserved for 1 year and 5 years, along with a 2.2-fold decrease in CD34^+^ cells in BM between fresh UCB and 1-year cryopreserved UCB (Figure 3, D and E), which was highly consistent with the tendency seen in the CFU assay.

Next, the secondary transplantation results revealed a dramatic decrease in reconstitution in the PB, BM, and SP of recipients, indicating that the impaired self-renewal capacity was more likely to be attributable to the cryopreservation process rather than the duration of the cryo-storage (Figure 3, F and G, and Supplemental Figure 4D). Moreover, there was no difference in the lineage reconstitution of myeloid and lymphoid cells (Myeloid/ B lymphoid/ T lymphoid (M/B/T) cells in BM; B/T/NK cells in SP) between the fresh and cryopreserved UCB CD34^+^ cells (Supplemental Figure 4, E and F). In agreement with the drastic transcriptomic variations that emerged within the 1-year cryopreservation period, the functional assays further demonstrated the short-term effect of cryopreservation on UCB-derived HSPCs. To further confirm the effects of the cryopreservation process on HSPCs, CD34^+^ HSPCs from cryopreserved UCB stored for 3 weeks were isolated for a CFU assay. Compared with the fresh UCB, the colony-forming ability was decreased for 3-week cryopreserved HSPCs (Supplemental Figure 4G). Consistently, we also observed a reduced colony-forming ability of cryopreserved CD34^+^ HSPCs collected from Shandong Qilu Stem Cell Engineering Co. (Supplemental Figure 4H).

To assess the correlation between the cryopreservation duration of UCB and clinical hematopoietic recovery, 171 patients with acute leukemia who underwent UCB transplantation with more than 3 × 10^5^ CD34^+^ cells/kg were analyzed (Supplemental Figure 4I and Supplemental Table 1). Apart from the positive correlation of the first complete response (CR1) and the granulocyte recovery previously described (25, 26), we observed a negative correlation between the median neutrophil engraftment time and UCB cryopreservation duration (Figure 3H), indicating the clinical ramification of UCB cryopreservation duration on hematopoietic recovery of recipients. Overall, the reconstitution of HSPCs from cryopreserved UCB gradually declined during the first 5 years of UCB cryostorage, but there was no additional decrease beyond this 5-year period, underscoring the effect of the cryopreservation process and the duration of cryopreservation.

### Decreased Mk production after cryopreservation.

To confirm the decreased capacity of cryopreserved HSC/MPP clusters to differentiate into Mks and erythrocytes predicted by FateID (Figure 2E and Supplemental Figure 3D), we performed single-cell and multi-cell (100 cells) liquid culture assays of the cells’ differentiation toward different lineages (27, 28). We found that fewer Mks were generated from cryopreserved HSPCs (Figure 3, I and J). Moreover, an in vivo assay using the NOD/ShiLtJGpt-Prkdc^em26Cd52^Il2rg^em26Cd22^kit^em1Cin(V831M)^/Gpt (NCG-X) mouse model 10 weeks after transplantation, which was more suitable for detecting megakaryocytic generation ability (29, 30), further validated the attenuated potential of cryopreserved HSPC differentiation toward Mks (Figure 3K). Notably, we did not detect human platelets in any of the mice given 10-year cryopreserved HSPCs until 7 weeks after transplantation, compared with the human platelet detection at 3 weeks in the fresh UCB group (Supplemental Figure 4J). In addition, the number of human platelets in the PB of recipients of cryopreserved HSPCs was decreased 10 weeks after transplantation compared with numbers in the fresh UCB group (Supplemental Figure 4K). However, both the in vitro colony-forming assays and in vivo transplantation experiments revealed a nonstatistically significant trend toward a reduced erythroid differentiation potential in cryopreserved HSPCs (Supplemental Figure 4, L and M), indicating that the erythroid lineage differentiation was mildly affected by cryopreservation. Overall, we observed that cryopreserved HSPCs had decreased potential of differentiation toward Mks and induced later platelet emergence, providing a possible explanation for the delayed platelet reconstitution observed in clinical UCB transplantation (31, 32).

### Little effect of cryopreservation on UCB-derived T and NK cells.

**Figure 4:**
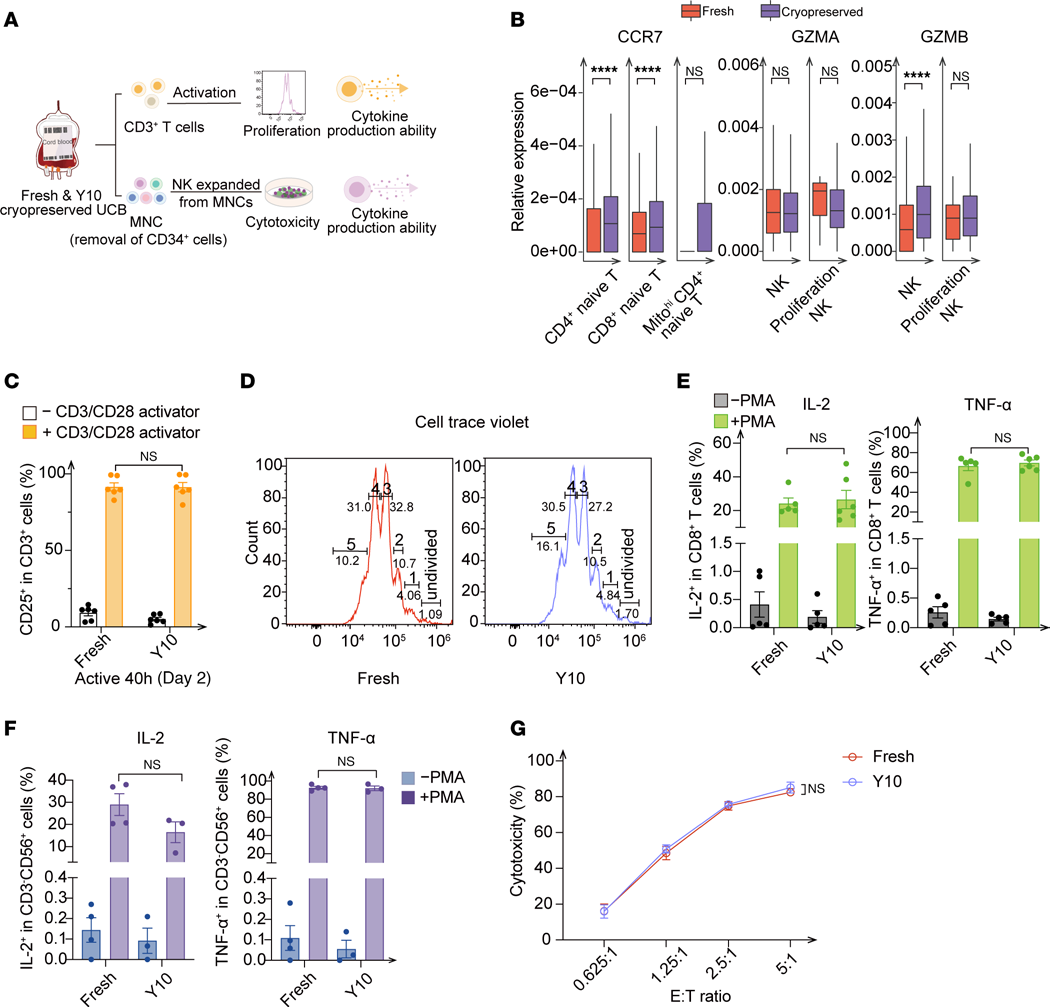
Minimal effect of cryopreservation on UCB-derived T and NK cells. (**A**) Experimental scheme to determine the effect of cryopreservation on T and NK cells (*n* = 4–6 replicates). (**B**) Relative expression levels of canonical naive (CCR7) and cytotoxicity-associated genes (*GZMA* and *GZMB*) in fresh and cryopreserved NK and T cells. ****P ≤ 0.0001, by Wilcoxon test. (**C**) CD25 expression in CD3^+^ T cells, with or without human CD3/CD28 activator, from fresh and cryopreserved UCB stored for 10 years (*n* = 6 replicates, 3 independent experiments). (**D**) Representative cell trace violet histograms of CD3^+^ T cells isolated from fresh and cryopreserved UCB stored for 10 years (*n* = 2 replicates, 4 independent experiments). (**E**) Expression of IL-2 and TNF-α in CD8^+^ T cells, with or without PMA and ionomycin, from fresh and cryopreserved UCB stored for 10 years (*n* = 5 replicates, 3 independent experiments). (**F**) The expression of IL-2 and TNF-α in expanded CD3^-^CD56^+^ cells, with or without PMA and ionomycin, from fresh and cryopreserved UCB stored for 10 years (*n* = 4 replicates, three independent experiments). (**G**) Cytotoxicity detection of expanded CD3^–^CD56^+^ cells from fresh and cryopreserved UCB stored for 10 years (*n* = 3 replicates, three independent experiments). Target cell: K562 cell lines. (**C** and **E**–**G**) Data indicate the mean ± SEM. Statistical significance was determined by 2-tailed *t* test.

Considering that Mito^hi^ subpopulations appeared among both HSPCs and mature blood cells after cryopreservation, we were interested in whether cryopreservation had an effect on the function of T and NK cells (Figure 4A), whose Mito^hi^ subpopulation accounted for a small proportion (Figure 1C). The expression levels of canonical naive and cytotoxicity-associated genes (C-C motif chemokine receptor 7 [*CCR7*], transcription factor 7 [*TCF7*], *CD27*, granzyme A [*GZMA*], granzyme B [*GZMB*], and killer cell lectin–like receptor D1 [*KLRD1*]) (33) in naive T and NK cell clusters after cryopreservation were not lower than those in the fresh group (Figure 4B and Supplemental Figure 5A), suggesting that cryopreservation had a minimal effect on the function of T and NK cells. More important, we observed no disparity in CD25 and CD69 expression levels induced by CD3/CD28 stimulation of the CD3^+^ T cells from fresh and cryopreserved UCB (Figure 4C and Supplemental Figure 5B). Furthermore, we found no evidence for cell division of CD3^+^ T cells or changes in the proportions of CD8^+^ subpopulations during the 7 days after stimulation in the cryopreservation groups (Figure 4D and Supplemental Figure 5, C–E). In particular, CD8^+^ T cells isolated from fresh and cryopreserved UCB produced comparable levels of IL-2 and TNF-α in the presence of phorbol myristate acetate (PMA) and ionomycin (Figure 4E), indicating similar cytokine production abilities of the T cells from UCB with and without cryopreservation.

The cytokine production ability and cytotoxic capacity of cryopreserved, UCB-expanded NK cells were similarly assessed (Figure 4A). Secretion levels of cytokines, including IL-2, TNF-α, and cytotoxic granules (GZMB and perforin) remained unaltered in expanded NK cells from cryopreserved UCB following PMA and ionomycin stimulation (Figure 4F and Supplemental Figure 5F). Given the direct recognition of tumor cells by NK cells (34), we cocultured expanded NK cells with K562 cells and found that NK cells derived from cryopreserved UCB displayed a cytotoxic capability comparable to that seen in cells derived from fresh UCB (Figure 4G). Collectively, the findings showed that cytokine production and cytotoxicity of T and NK cells were unaffected by cryopreservation, demonstrating the diversity of different UCB compartments in the responses to cryopreservation.

### Mitochondrial dysfunction leads to attenuated hematopoietic reconstitution in cryopreserved, UCB-derived HSPCs.

**Figure 5:**
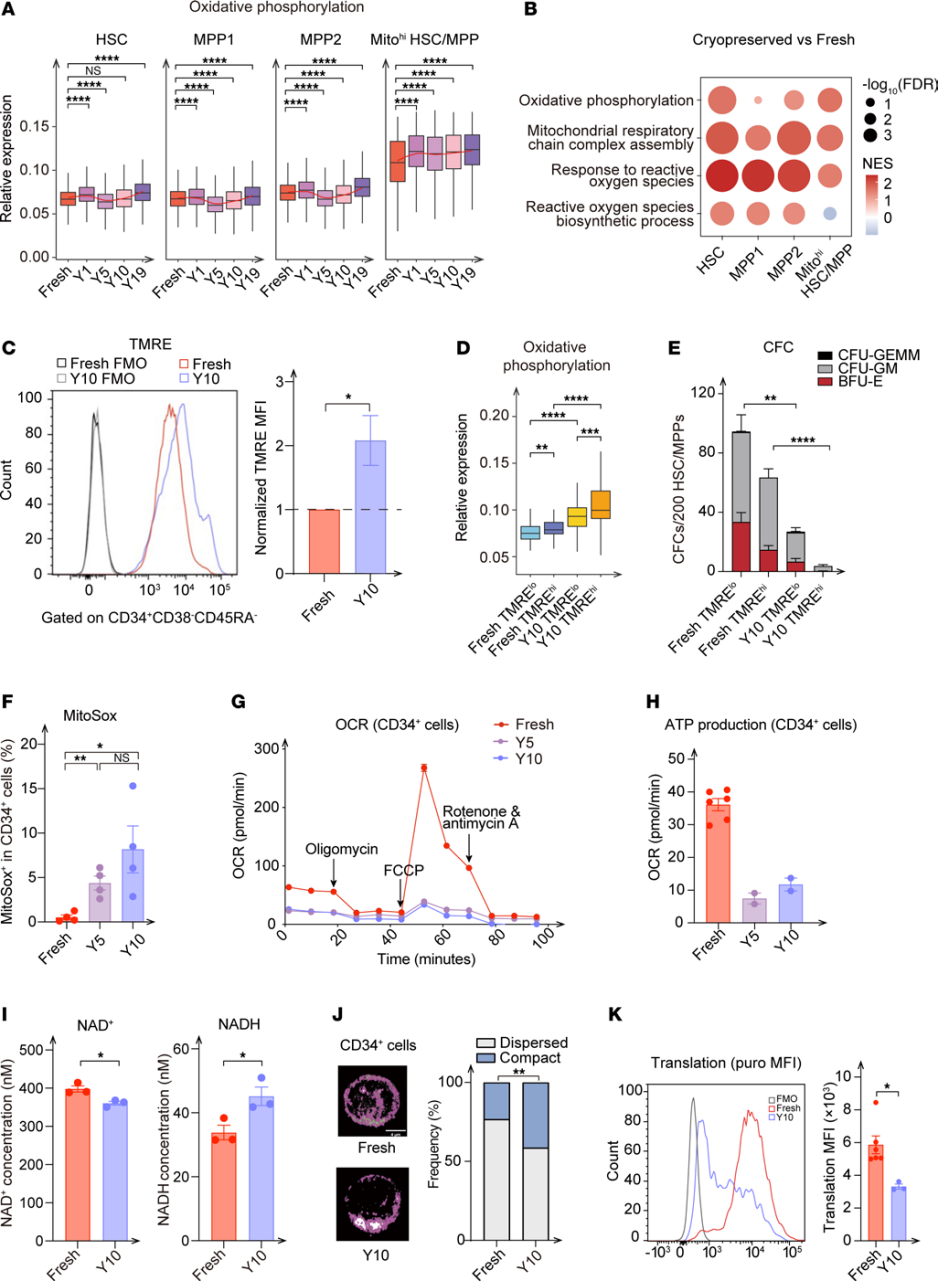
Reduced hematopoietic reconstitution is attributed to abnormal mitochondrial metabolism. (**A**) Relative expression of OXPHOS genes in HSCs, MPP1s, MPP2s, and Mito^hi^ HSCs/MPPs from fresh and cryopreserved UCB. ****P < 0.001, by Wilcoxon test to compare each cryopreserved group with fresh UCB. (**B**) Bubble plot showing the upregulated gene sets in cryopreserved UCB HSC/MPP clusters, using GSEA to calculate the normalized enrichment score (NES). Cryopreserved UCB versus fresh UCB, with UCB from different cryopreservation years regarded as a whole. (**C**) Histogram and normalized TMRE MFI of HSCs/MPPs from fresh and cryopreserved UCB (10 years) (*n* = 2 replicates, 2 independent experiments). (**D**) Relative expression of OXPHOS genes in HSCs/MPPs with low and high TMRE signal from fresh and 10-year cryopreserved UCB. **P ≤ 0.01, ***P ≤ 0.001, and ****P ≤ 0.0001, by Wilcoxon test. (**E**) CFCs per 200 HSCs/MPPs from fresh and 10-year cryopreserved UCB, stratified by TMRE signal (*n* = 5 replicates). (**F**) Percentage of MitoSox^+^ cells in CD34^+^ population from fresh and 5-year and 10-year cryopreserved UCB (*n* = 4 replicates). *P ≤ 0.05 and **P ≤ 0.01, by 2-tailed t test. (**G** and **H**) OCR (**G**) and ATP production (**H**) of CD34^+^ cells from fresh and 5- and 10-year cryopreserved UCB (fresh, *n* = 3 pooled UCB, 6 technical replicates; 5 years, *n* = 4 pooled UCB, 2 technical replicates; 10 years, *n* = 4 pooled UCB, 2 technical replicates). (**I**) NAD^+^ and NADH levels in fresh UCB and 10-year cryopreserved UCB HSPCs. NAD^+^ or NADH concentration: nM/2 × 10^6^ cells. (**J**) Representative immunofluorescence confocal image of TOM20 expression and percentages of dispersed and mitochondrial network analysis showing dispersed versus compact mitochondria in CD34^+^ cells from fresh (90 cells) and 10-year cryopreserved UCB (104 cells). **P ≤ 0.01, by Fisher’s exact test. (**K**) Representative histogram and translation level (anti-puro MFI) of CD34^+^ cells from fresh and 10-year cryopreserved UCB. *P ≤ 0.05, **P ≤ 0.01, and ****P ≤ 0.0001, by 2-tailed *t* test (**C**, **E**–**I**, and **K**). Data indicate the mean ± SEM.

The discovery of HSC/MPP-specific functional attenuation after cryopreservation prompted us to clarify the underlying mechanisms. Considering the increased abundance of Mito^hi^ HSCs/MPPs (Figure 1E) and the unsupervised GO enrichment results for the cryopreservation samples (Figure 2B), we first confirmed the upregulation of oxidative metabolism gene sets in the HSC/MPP clusters represented by Mito^hi^ HSCs/MPPs after cryopreservation (Figure 5, A and B). Interestingly, unlike other Mito^hi^ subpopulations, oxidative metabolism was not transcriptionally activated in Mito^hi^CD4^+^ naive T cells after cryopreservation (Supplemental Figure 6A), which might explain why T cells were less affected by cryopreservation. As indicated by the elevated tetramethylrhodamine ethyl ester (TMRE) signals, the mitochondrial membrane potential of HSCs and MPPs from cryopreserved UCB was increased (Figure 5C). To ascertain whether increased TMRE levels gave rise to impaired hematopoietic reconstitution, HSCs/MPPs with low and high TMRE signal levels from fresh and cryopreserved UCB were sorted to conduct single-cell tagged reverse transcription RNA-Seq (STRT-Seq) and a CFU assay. We detected higher expression levels of mitochondrial metabolism genes (Figure 5D and Supplemental Figure 6, B–D) and lower colony-forming ability (Figure 5E) in cryopreserved HSC and MPP populations with higher TMRE signals than in those with lower signals. These results implied that higher oxidative metabolism levels correlated to lower hematopoietic reconstitution in cryopreserved HSPCs.

To further explore the impairment mechanisms, we measured mitochondrial ROS levels by MitoSox test and found them to be markedly higher in the CD34^+^ cells from UCB samples that had been cryopreserved for 5 and 10 years (Figure 5F and Supplemental Figure 6E). Indeed, mitochondrial ROS levels were elevated as early as 3 weeks after cryopreservation (Supplemental Figure 6F). Previous studies have substantiated that mitochondrial ROS levels were closely associated with oxidative respiration and ATP synthesis (35, 36). We therefore measured their energy metabolism levels and found a reduction in the oxygen consumption rate (OCR) and ATP production of cryopreserved HSPCs (Figure 5, G and H), but there was no significant difference in their extracellular acidification rate (ECAR) (Supplemental Figure 6G). Moreover, compared with CD34^+^ cells from fresh UCB, cryopreserved HSPCs exhibited decreased nicotinamide adenine dinucleotide (NAD^+^) levels and increased nicotinamide adenine dinucleotide (reduced form, NADH) levels, indicating a decline in mitochondrial respiratory chain activity in cryopreserved UCB HSPCs (Figure 5I). Additionally, we revealed that fresh CD34^+^ cells were more likely to exhibit dispersed mitochondria (more individual mitochondria), whereas more compact mitochondria (more mean branches per network) were prevalent in cryopreserved HSPCs (Figure 5J and Supplemental Figure 6H). In contrast, the OCR was not reduced in CD3^+^ T cells from UCB cryopreserved for 10 years, providing a further explanation for the lesser effect of cryopreservation on T cells (Supplemental Figure 6I). Finally, we observed a reduction in protein synthesis in cryopreserved CD34^+^ cells compared with the fresh compartment (Figure 5K), which may help explain the paradoxical observation of high MitoSox staining, increased TMRE, and elevated expression of oxidative phosphorylation–related (OXPHOS-related) genes, despite lower OCRs. Collectively, our findings provide evidence that the reduced hematopoietic reconstitution of cryopreserved UCB can probably be attributed to dysfunctions in mitochondrial metabolism, including higher levels of OXPHOS and ROS, reduced oxygen consumption coupled with reduced ATP production, and changes in mitochondrial distribution.

### Intervention strategy to improve the function of cryopreserved, UCB-derived HSPCs.

**Figure 6:**
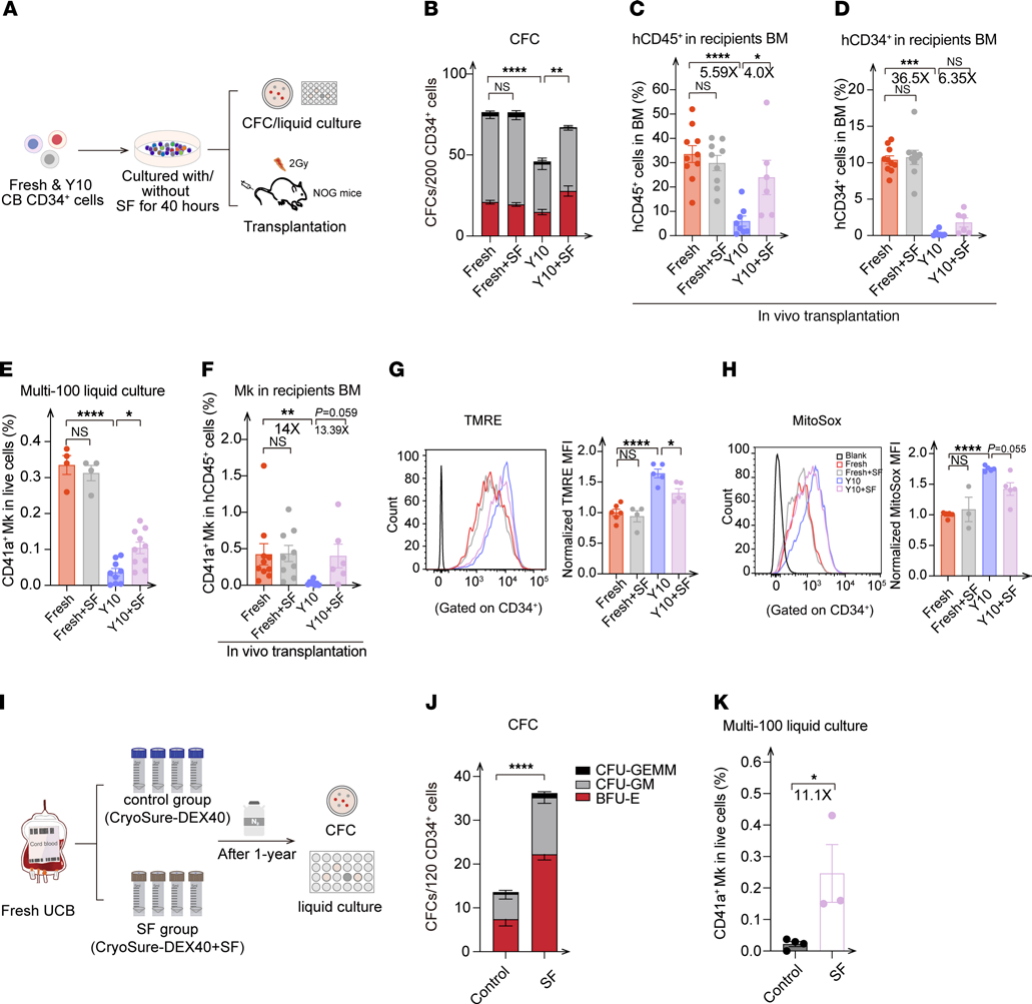
Impaired function of cryopreserved, UCB-derived HSPCs can be ameliorated by SF. (**A**) Experimental scheme for analysis of cryopreserved HSPCs treated with SF. CD34^+^ cells from cryopreserved UCB were cultured with or without SF, followed by FACS for CFU, liquid culturing, and transplantation assays. For transplantation, UCB-derived CD34^+^ cells (20,000/mouse) were injected into mice via the tail vein. (**B**) CFCs per 200 CD34^+^ cells from fresh and cryopreserved UCB stored for 10 years, cultured with or without SF treatment (*n* = 5 replicates, 3 independent experiments). (**C** and **D**) Percentage of hCD45^+^ and hCD34^+^ cell engraftment in BM of recipients 12 weeks after transplantation (*n* = 6–10 recipient mice per group). (**E**) Lineage output of Mks (CD41a^+^) from bulk (100 cells) CD34^+^ cells from fresh and 10-year cryopreserved UCB with or without SF treatment (*n* = 3–4 pooled UCB, 2 independent experiments). (**F**) Percentage of human CD41a^+^ cell engraftment in BM of recipients 12 weeks after transplantation. (**G**) Representative histogram and normalized TMRE MFI of CD34^+^ cells from fresh and 10-year cryopreserved UCB cultured with or without SF (*n* = 3–5 replicates, 2 independent experiments). (**H**) Representative histogram and normalized MitoSox MFI of CD34^+^ cells from fresh and 10-year cryopreserved UCB cultured with or without SF (*n* = 4–6 replicates, 2 independent experiments). (**I**) Experimental scheme for cryopreservation with or without 10 μM SF added to the freezing solution (control: *n* = 6 replicates; SF: *n* = 6 replicates). (**J**) CFCs per 120 CD34^+^ cells from control and SF-treated groups (*n* = 5 replicates). (**K**) Lineage output of Mks (CD41a^+^) from bulk (100 cells) CD34^+^ cells from control and SF-treated groups (*n* = 3–4 replicates). All data indicate the mean ± SEM. *P ≤ 0.05, **P ≤ 0.01, ***P ≤ 0.001, and ****P ≤ 0.0001, by 1-way ANOVA or Kruskal-Wallis test, with a post hoc Tukey’s test applied for multiple comparisons (**B**–**H**) and 2-tailed *t* test (**J** and **K**).

Facing the oxidative metabolism dysregulation of cryopreserved HSPCs, we therefore attempted to improve hematopoietic reconstitution and eliminate the excessive mitochondrial ROS by using antioxidant interventions after UCB thawing (Figure 6A). We screened various antioxidants, e.g., sulforaphane (SF), *N*-acetylcysteine (Nac), resveratrol, vitamin E, and coenzyme Q10. We found that both Nac and SF treatment increased the colony-forming ability of cryopreserved CD34^+^ cells in vitro. However, the effects of Nac were less pronounced than those of SF, leading us to select SF for further experiments (Supplemental Figure 7A). SF, an isothiocyanate derived from cruciferous vegetables (37–39), alleviated the bystander effects induced by radiation, as demonstrated in our previous work (38). Here, we observed a pronounced enhancement in the clonogenic potential of cryopreserved CD34^+^ cells following 40 hours of culturing with SF compared with cells cultured without SF. However, SF treatment had a minimal effect on the colony-forming capacity of fresh samples (Figure 6B). This observation was further supported by in vivo functional assays, which showed an increase in the engraftment of human CD45^+^ (hCD45^+^) cells and CD34^+^ cells in the BM of recipients transplanted with cryopreserved UCB cultured with SF compared with those cultured without SF, following long-term transplantation (Figure 6, C and D, and Supplemental Figure 7, B and C). Moreover, the lymphoid and myeloid lineage reconstitution abilities of SF-treated and untreated groups (M/B/T cells in BM and B/other cells in SP) in recipient mice were not significantly different (Supplemental Figure 7, D and E). We further assessed whether the reduced Mk production after cryopreservation could also be improved by SF treatment. We found that the in vitro Mk differentiation ability of HSPCs cryopreserved for 10 years increased considerably in the SF treatment group and was even comparable to that of the fresh UCB group, as indicated by the multi-cell (100 cells) liquid culture result (Figure 6E), which was further confirmed by the elevated Mk and platelet levels in BM and PB, respectively, from recipient mice after transplantation (Figure 6F and Supplemental Figure 7, B and F). Moreover, we found that SF significantly reduced both the mitochondrial membrane potential and ROS levels in cryopreserved CD34^+^ cells stored for 10 years (Figure 6, G and H), potentially through its antioxidant activity.

Considering the clinical procedure of direct reinfusion of post-thaw UCB into patients, we tried to add SF to the UCB before cryopreservation to determine whether this would have similar protective effects (Figure 6I). After 1 year of cryopreservation, we observed an increase in the clonogenic potential of cryopreserved CD34^+^ cells from the SF-treated group compared with the control group (Figure 6J and Supplemental Figure 7G). Concurrently, a multi-cell (100 cells) liquid culture assay showed that the Mk differentiation ability of cryopreserved CD34^+^ cells was substantially heightened with SF treatment in comparison with that of the control group (Figure 6K). Additionally, we also observed a reduction in mitochondrial ROS levels in the SF-treated group compared with the control group (Supplemental Figure 7H). These findings indicated that SF treatment can be used to ameliorate the adverse effects of cryopreservation on HSPC reconstitution and Mk production, providing an effective strategy to facilitate the broader application of long-term cryopreserved UCB in clinical transplantation.

## Discussion

Our results indicated diverse effects of cryopreservation on distinct cell populations in UCB. In contrast to mature T and NK cells, which maintained normal biological function after cryopreservation, HSPCs featured decreased expression levels of HSC/MPP signature genes and a gradual reduction in long-term engraftment, which stabilized after 5 years of storage. In addition, there was a notable decrease in the self-renewal capacity during the short-term cryopreservation periods (1 year). Although single-variable analysis did not identify a substantial effect of UCB cryopreservation duration on hematopoietic recovery (40), our clinical data revealed a correlation between the increased UCB cryopreservation duration and delayed neutrophil engraftment after we corrected for covariates including age, CR1, HLA mismatch, and other factors. Additionally, we noted a diminishment of the capacity of cryopreserved HSPCs to differentiate toward Mks, providing a potential rationale for the delayed platelet production in the clinical transplantation of UCB (31, 32). Because the platelet engraftment would be influenced by the platelet transfusion after transplantation in the clinic, we could not analyze the correlation of between the cryopreservation duration of UCB and platelet recovery. These temporal alterations to HSPC functions emphasized the combined effects of the initial cryopreservation process and the duration of cryopreservation.

A recent study revealed that cryopreserved UCB stored for 27 years (27yoCB) was still highly functional by comparison with previously described fresh UCB (21). However, in our study, although UCB-derived HSPCs cryopreserved for up to 19 years also maintained the capacity for prolonged reconstitution, aligning with the authors’ perspective, the long-term reconstitution and self-renewal ability of cryopreserved UCB–derived HSPCs were attenuated compared with the parallel fresh control. Our seemingly debated results were not contradictory to their study: their study predominantly highlighted the engraftment potential of 27yoCB, whereas our investigation heightened the clinical relevance by incorporating parallel comparisons and quantitative assessments of the transcriptomic and functional dynamics across various cryopreservation durations (fresh, 1, 5, 10, and 19 years), revealing a nonlinear functional decline after cryopreservation. Therefore, the clinical value of our study may suggest that the long-term cryopreserved UCB units, especially those stored for more than 5 years, up to 19 years, should not be excluded when choosing suitable UCB samples.

Increasing evidence shows the importance of mitochondrial metabolic balance in maintaining the function of HSCs (35, 36, 41, 42). With the appearance of specific Mito^hi^ subpopulations in various cell types, HSPCs in particular exhibited dysfunctional mitochondrial metabolism after cryopreservation, as evidenced by increased oxidative phosphorylation levels, higher ROS levels but a lower OCR, reduced ATP production, and mitochondrial distribution changes. Importantly, these types of damage induced by the dysregulated mitochondrial metabolism in cryopreserved HSPCs (even if stored for 10 years) were efficiently ameliorated by SF. It has been reported that SF can upregulate the expression of genes that fight against oxidative stress, inflammation, DNA-damaging electrophiles, and radiation damage, and inhibit breast cancer stem cells (38, 39, 43). As a chemopreventive agent, SF can be efficiently and rapidly absorbed within the human small intestine and has remarkable bioavailability and minimal toxicity (39, 43, 44). Hence, in light of our research results, we believe that SF has immense potential for ameliorating cryopreservation-induced mitochondrial dysregulation by such actions as reducing ROS levels and enhancing hematopoietic recovery after UCB transplantation.

When faced with same cryopreservation stress severity and duration, T and NK cells seem to be more resistant than HSPCs, as shown by the comparable functions between fresh and cryopreserved UCB groups. In contrast to the Mito^hi^ HSCs/MPPs, oxidative metabolism–related genes were not transcriptionally activated in Mito^hi^CD4^+^ naive T cells after cryopreservation. Functional assays also revealed that the oxygen consumption ability of T cells from cryopreserved UCB was not affected. In addition, prior studies indicated that downregulated expression levels of pluripotency markers caused by cryopreservation were not universal across different stem cell types (45). All of these findings help to explain why T and NK cells are more resistant to cryopreservation. These phenomena highlight the heterogeneous effects of cryopreservation on the diverse cell types found in UCB. Considering the functional integrity of cryopreserved T and NK cells, UCB-derived immune cells collected at birth could be used to treat future immune deficiencies or disease, especially via individualized treatments, and could also prove to be a valuable resource for CAR-T and CAR-NK therapies.

In conclusion, our work involved a comprehensive evaluation of the functions of different cell populations in cryopreserved UCB through a side-by-side comparison with fresh UCB cells. Notably, the emergence of Mito^hi^ cell populations induced by cryopreservation was not limited to UCB. Similar cell populations have been observed in PB and BM (data not shown), suggesting that this phenomenon may be a characteristic of different blood samples. As such, the precise effects of cryopreservation on distinct types of blood samples and diverse cell populations warrant further investigation. We propose a viable improvement strategy to ameliorate attenuated hematopoietic reconstitution and delayed platelet production and demonstrate that T and NK cells were largely unaffected by cryopreservation. This information provides strong foundations for the broader application of UCB, which will benefit more patients with hematologic and immunologic diseases.

## Methods

### Sex as a biological variable.

Animal studies were conducted using female mice, as sex differences in hematopoietic reconstitution in recipients have been reported (46). However, on the basis of our human study results, we expect the findings to be relevant regardless of biological sex.

### Data sources.

Single-cell transcriptomics data on live CD34^+^ HSPCs and MNCs derived from fresh (*n* = 4 single-donor UCB) and cryopreserved UCB stored for 1, 5, 10, or 19 years (*n* = 4 single-donor UCB for each year) were generated individually by 10x Genomics. In a replicate experiment, CD34^+^ HSPCs and MNCs isolated from UCB cryopreserved for 10 years (*n* = 4 single-donor UCB) were obtained from Shandong Qilu Stem Cell Engineering Co. and were also submitted to 10x Genomics. UCB samples for transcriptome analysis and experimental validation were obtained from the Blood Biobank of Institute of Hematology and Blood Diseases Hospital and Tianjin Cord Blood Bank. All cryopreserved samples were processed using the same method and preserved in identical media.

### Sample preparation.

Fresh UCB was subjected to processing within a temporal window spanning 12–24 hours following parturition. Cryopreserved UCB samples were thawed in a temperature-controlled water bath at 37°C. After thawing, UCB samples were diluted 1:1 with PBS, and the isolation of MNCs was achieved by density-gradient centrifugation using Histopaque-1077 (MilliporeSigma). After this, cell viability was tested with the TC20 Automated Cell Counter (Bio-Rad). Subsequently, lineage^+^ cells were depleted using the EasySep Human Progenitor Cell Enrichment kit with the Platelet Depletion kit (Stemcell Technologies). Lineage-depleted cells were stained with anti-human antibodies, including a lineage cocktail (CD3, CD14, CD16, CD19, CD20, CD56; BV510, BioLegend); CD34-APC (clone 581, BD Biosciences); CD38-FITC (clone HB7, BD Biosciences); CD45RA-APC/Cy7 (clone HI100, BioLegend); CD90-PerCP/Cyanine 5.5 (clone 5E10, BD Biosciences); CD10-BV786 (clone HI10a, BD Biosciences); CD135-PE (clone 4G8, BD Biosciences), and Brilliant Stain Buffer (BD Biosciences). Samples underwent a 30-minute incubation on ice in the absence of light, followed by a thorough washing using PBS plus 2% FBS. Subsequently, cellular suspensions were supplemented with DAPI (1 μg/mL, MilliporeSigma) to facilitate the removal of nonviable cells. Live cells were sorted with a BD FACSAria III instrument from BD Bioscience.

### Animal model.

For primary and secondary xenotransplantations, 8-week-old female NOD/Shi-SCID/IL2Rg^null^ (NOG) mice or NCG-X mice, purchased from the Institute of Laboratory Animals, Chinese Academy of Medical Sciences, were used in this study. The mice were raised in sterile mouse cages in specific pathogen–free rooms with continuous positive air pressure. The mouse bedding, food, and drinking water underwent sterilization through high-temperature and pressure treatment.

### Clinical study design and patients.

We conducted a retrospective analysis encompassing 701 consecutive patients with acute leukemia, who were treated between 2015 and 2020. All patients underwent unrelated single-unit UCBT with myeloablative conditioning (MAC) without antithymocyte globulin at the First Affiliated Hospital of University of Science and Technology of China. Considering that the CD34^+^ infusion dosage is a crucial factor influencing the engraftment of neutrophils in recipients in a highly nonlinear manner (25, 26, 47), patients with an infused CD34^+^ cell dosage of 3.0 × 10^5^ cells/kg or less (*n* = 529) and those who died before neutrophil engraftment (*n* = 1) were excluded. Our cohort ultimately included 171 individuals. The patient selection flowchart is provided in Supplemental Figure 3H.

### Multivariate quantile regression.

To correct for confounding factors, we conducted quantile regression analysis to quantify the independent effect of cryopreservation duration on the median neutrophil engraftment time when other factors such as years at transplantation, leukemia state, HLA mismatch in GVHD direction, etc., were included as the covariates (*P ≤ 0.05) (Supplemental Table 1).

### Library construction and sequencing.

HSC and MPP populations (CD34^+^CD38^–^CD45RA^–^) with low and high TMRE signal levels were obtained from fresh and cryopreserved UCB stored for 10 years and sorted before STRT-Seq was performed, as our previous work describes (28). Single-cell RNA libraries of HSPCs and MNCs from all UCB samples were constructed with a KAPA Hyper Prep kit (Kapa Biosystems), and sequencing was achieved using the Illumina HiSeq 4000 platform, as paired-end 150-bp reads (Novogene).

### Processing of single-cell RNA-Seq data.

To obtain the unique molecular identifier (UMI) count from the STRT-Seq data, raw sequencing data were demultiplexed by Python scripts, followed by cleaning, alignment, and calculation of the reads, as described in our previous work (28). For 10x Genomics, single-cell expression matrices for all UCB samples were generated in Cellranger (version 6.1.2) with the “count” function and with reference to the GRCh38 genome. First, considering the higher quality of CD34^+^ cells compared with MNCs, only those CD34^+^ cells satisfying the criteria of (a) 500<total gene number<6,000; (b) 1,000<total UMI count<40,000; and (c) percentages of mitochondrial UMIs<15%; and only those MNCs satisfying the criteria of (a) 500<total gene number<4,000; (b) 1,000<total UMI count<20,000; and (c) percentages of mitochondrial UMIs<15%, excluding the doublets evaluated by Scrublet (48) (version 0.2.1) with default parameters, were retained for further analysis. Since our study focused on mitochondrial subclusters, we applied a relatively stringent percentage of mitochondrial UMIs of less than 15%. Meanwhile, to predict the hematopoietic cell types in the UCB, the UMI matrix for each sample was projected onto the ABC by “TransferData” from Seurat (49) (version 4.0.3). Next, the high-quality UMI matrices for 40 samples (covering CD34^+^ cells and MNCs from fresh and cryopreserved UCB stored for 1, 5, 10, or 19 years) were integrated by Scanpy (version 1.5.1) (50). This was followed by normalization, logarithmization, highly variable gene (HVG) detection, and regressing out of the cell-cycle and scale. Subsequently, principal component analysis (PCA) dimension reduction and batch-effect correction by the BBKNN algorithm were performed using Scanpy (version 1.5.1), and the resulting data were submitted for UMAP dimension reduction, clustering by the Leiden algorithm, and UMAP visualization. Finally, according to the predicted cell types projected onto ABC and canonical signature genes, 143,385 HSPCs, 79,938 T/NK cells, 22,938 B cells, and 36,844 monocytes were precisely identified.

To further resolve the heterogeneity, the UMI matrices of HSPCs, T/NK cells, B cells, and monocytes were respectively extracted for reprocessing and reclustering by Seurat (version 4.0.3), including normalization by “NormalizeData,” HVG detection by “FindVariableFeatures,” integration by “IntegrateData,” scale by “ScaleData,” and dimension reduction by “RunPCA” and “RunUMAP,” along with clustering by “FindNeighbors” and “FindClusters.” Eventually, 12 cell clusters for HSPCs, 8 cell clusters for T/NK cells, 6 cell clusters for B cells, and 8 cell clusters for monocytes were determined for analysis. Moreover, Mito^hi^ subpopulations were defined by elevated mitochondrial gene expression with high gene numbers and UMI counts (for example, 2,462 genes and 8,019 UMI counts for Mito^hi^ HSCs/MPPs), which were markedly different from those poor-quality cells with low gene numbers and UMI counts of less than 500 and 1,000, respectively. The 10-year cryopreserved UCB samples obtained from Shandong Qilu Stem Cell Engineering Co. were processed as described above, and the high-quality UMI matrices for 16 samples (covering CD34^+^ cells and MNCs from fresh and cryopreserved UCB) were integrated by Scanpy (version 1.5.1). This was followed by the aforementioned procedures to achieve the identification of hematopoietic cell types. Additionally, FateID (24) was used to assess the potential of HSC/MPP differentiation into 3 lineages.

### Identification of signature genes and transcriptomic differences.

“FindAllMarkers” from Seurat (version 4.0.3) was used to determine cell cluster–specific signature genes, whereas “FindMarkers” was applied to detect DEGs between any 2 given groups. The filtered criteria for signature genes and DEGs was a fold change of 1.5 or more and an adjusted *P* value of 0.05 or less. The number of DEGs was used to evaluate the degree of transcriptomic variation in each cell cluster between any 2 years. GO enrichment analysis for DEGs was performed using Metascape (51) and clusterProfiler (52), while gene set enrichment analysis (GSEA) was used to examine the statistical significance of gene activation for specific biological processes.

### Calculation of the fraction of UMIs.

To compare the relative expression levels of HSC/MPP signature and oxidative metabolism genes between fresh and cryopreserved groups, their UMI fractions in corresponding cell clusters were calculated as the total UMIs of gene sets divided by the total UMIs of each single cell (53). HSC/MPP signature genes were the top 50 signature genes of HSC/MPP clusters, whereas oxidative metabolism terms were downloaded from the GSEA website.

### Cell-cycle analysis.

Single cells were designated as being in the G_1_, S, or G_2_M phase according to the S Score and G_2_M score calculated by Seurat (version 4.0.3), based on 43 genes associated with G_1_/S and 55 genes related to G_2_/M summarized from a previous study (54).

### CFU assay.

CD34^+^ cells from fresh UCB and UCB cryopreserved for different durations were individually sorted into methylcellulose-based medium (H4034, Stemcell Technologies). This medium was supplemented with human IL-6 (hIL-6) (50 ng/mL, Peprotech) and human Fms-related tyrosine kinase 3 (hFLT-3) (20 ng/mL, Peprotech). Subsequently, the plating procedure was performed in nontreated, 24-well culture plates according to the manufacturer’s protocol (Stemcell Technologies). Following an incubation period of 12–14 days under 5% CO_2_ and a temperature of 37°C, colonies were categorized and calculated using an inverted optical microscope.

### Single- and multi-cell liquid culturing.

Single- and multi-cell liquid culturing was performed as previously described by Zhang et al. (28). Two days prior to liquid culturing, untreated 96-well plates or 24-well plates were precoated with 40 μL or 400 μL 0.1% gelatin solution (Stemcell Technologies) at a temperature of 4°C, and this coating was maintained overnight. Then, the gelatin was removed, and murine MS-5 stromal cells were seeded at a concentration of 3 × 10^4^ cells/mL using 100 μL H5100 medium (Stemcell Technologies) in 96-well plates, or 1 mL H5100 medium in 24-well plates. After 24 hours, the H5100 medium was replaced with 200 μL erythro-myeloid differentiation medium containing StemPro-34 SFM medium (Thermo Fisher Scientific) supplemented with the following cytokines (all from Peprotech): SCF (100 ng/mL); FLT-3L (20 ng/mL); TPO (100 ng/mL); IL-6 (50 ng/mL); IL-3 (10 ng/mL); IL-11 (50 ng/mL); GM-CSF (20 ng/mL); IL-2 (10 ng/mL); IL-7 (20 ng/mL); and EPO (3 units/mL), as well as 1% penicillin/streptomycin (Gibco, Thermo Fisher Scientific) and 1% l-glutamine (Gibco, Thermo Fisher Scientific). Then, single- and multi-cell (100 cells) cultured CD34^+^ cells were sorted into each well and cultured with 5% CO_2_ at 37°C. After 2 weeks, the cells were harvested and subjected to flow cytometric analysis. Each colony was stained with the following antibodies: CD45-APC/Cy7 (clone 2D1, BD Bioscience); CD14-PC7 (clone RM052, Beckman Coulter); CD15-FITC (cloneHI98, BioLegend), CD235a-PE (clone 11E4B-7-6, Beckman Coulter); CD41a-APC (clone HIP8, BD Bioscience); and CD56-PerCP/Cyanine 5.5 (clone B159, BD Bioscience). Myeloid cell colonies were assessed and defined as containing 30 or more CD45^+^CD14^+^ or CD45^+^CD15^+^ cells; Mk colonies as 10 or more CD41a^+^ cells; erythrocyte colonies as 30 or more (CD45^–^CD235a^+^) cells; and NK cell colonies as 30 or more (CD45^+^CD56^+^) cells.

### Transplantation assays.

UCB samples were subjected to pooling (4–6 samples per pool) before transplantation. For primary xenotransplantation, 8- to 12-week-old female NOG mice were subjected to 250 cGy sublethal irradiation 24 hours prior to transplantation, and 8- to 12-week-old female NCG-X mice were subjected to 50 cGy irradiation. Equal numbers of CD34^+^ cells (10,000–50,000/mouse, the specific cell numbers for each experiment were available within the associated legends) were injected via the tail vein. To monitor engraftment and human platelet production, PB samples were obtained at 4, 8, 12, 16, and 20 weeks after transplantation. After 16–20 weeks, mice were sacrificed to obtain BM and SP for analysis. Human chimerism was evaluated through flow cytometric analysis utilizing the following antibodies (unless otherwise specified, all antibodies were obtained from BD Biosciences): anti–mouse CD45-PerCP/cyanine 5.5 (clone 30-F11); anti–mouse CD45-APC/Cy7 (clone 30-F11); anti–human CD45-APC/Cy7 (clone 2D1, CD45-PE/Cy7 (clone HI30); CD33-APC (clone P67.6); CD19-PE (clone 4G7); CD14-PC7 (clone RM052, Beckman Coulter); CD15-V450 (clone MMA); CD235a-PE (clone 11E4B-7-6, Beckman Coulter); CD41a-APC (clone HIP8); CD3-FITC (clone UCHT1); and CD56-PerCP/Cyanine 5.5 (clone B159). For secondary transplantation, 1 × 10^7^ cells from each primary recipient were collected following the above methods. Twelve weeks after transplantation, the mice were euthanized, and their BM and SP cells were collected for subsequent analysis.

### Apoptosis assay.

The isolated CD34^+^ cells were washed twice with cold PBS. Following this, they were resuspended with 100 μL 1× binding buffer and incubated with 5 μL annexin V and 10 μL propidium iodide (PI) staining solution for 15 minutes at room temperature according to the manufacturer’s protocol (Yeasen). After that, an additional 400 μL 1× binding buffer was added to the samples. The assessment of apoptotic cell proportions was carried out via flow cytometric analysis.

### Membrane potential analyses and mitochondrial ROS.

UCB CD34^+^ cells were isolated by positive selection with the CD34 Microbead kit following the manufacture’s guidelines (Miltenyi Biotec). Following surface marker staining with CD34-APC (clone 581, BD Biosciences); CD38-FITC (clone HB7, BD Biosciences); CD45RA-APC/Cy7 (clone HI100, BioLegend); and CD90-PerCP/Cyanine 5.5 (clone 5E10, BD Biosciences), the cells were incubated in HBSS at 37°C for 20 minutes with MitoSox Red (Invitrogen, Thermo Fisher Scientific) at a final concentration of 2.5 μM or with TMRE (MilliporeSigma) at a final concentration of 0.1 μM (55). Thereafter, the cells were washed with HBSS, labeled with DAPI, and analyzed and sorted with the BD FACSAria III.

### Seahorse OCR and ECAR measurements.

Extracellular acidification rate (ECAR) and OCR measurements were performed utilizing the Seahorse xFe24 analyzer (Seahorse Bioscience). The isolated UCB CD34^+^ cells were washed and suspended in prewarmed Seahorse XF DMEM medium containing 25 mM glucose, 1 mM pyruvate, and 5 mM glutamine (38, 56). The cells were attached to the bottom of a xFe24 Tissue Culture Plate (Agilent Technologies) at a density of 5 × 10^5^ cells per well coated with BD Cell-Tak Cell Adhesive (Corning). Cell mitochondrial stress was assessed under basal conditions in the presence of 1 μM oligomycin, 5 μM carbonyl cyanide *p*-trifluoro methoxyphenylhydrazone (FCCP), and 1 μM rotenone and antimycin A (ROT/AA) according to the manufacturer’s instructions. Additionally, a glycolytic rate assay was conducted under basal conditions in the presence of 0.5 μM ROT/AA and 50 mM 2-deoxy-D-glucose (2-DG). Data analysis was performed using the Agilent Seahorse Glycolytic Rate Assay Report Generator.

### Immunofluorescence.

UCB CD34^+^ cells were sorted and fixed with 4% paraformaldehyde at room temperature for 8 minutes and permeabilized with 0.5% Triton X-100 for 15 minutes, followed by blocking in 5% BSA with 0.1% Triton X-100 plus PBS for 1 hour. The samples were then stained with primary mouse anti–translocase of the outer mitochondrial membrane 20 (TOM20) antibody at 1:100 dilution (sc-17764, Santa Cruz Biotechnology) overnight at 4°C. The secondary antibody Alexa Fluor 488 rabbit anti–mouse IgG^(H^^+^^L)^ (1:100 dilution, A27023, Invitrogen, Thermo Fisher Scientific) was applied at room temperature for 1 hour. Finally, the samples were processed for nuclear staining using DAPI. Image acquisition was carried out using a 100× lens objective with Z stacks for comprehensive visualization. Mitochondrial morphology of CD34^+^ cells from fresh and cryopreserved UCB stored for 10 years was analyzed using the plug-in macro function MiNA (Mitochondrial Network Analysis), which was used for semiautomated analysis of mitochondrial networks in cells (57–59).

### T cell activation and cell division tracking.

UCB CD3^+^ cells were isolated by positive selection with the CD3 Microbead kit (Miltenyi Biotec), following the manufacturer’s guidelines. Purified CD3^+^ T cells were labeled with 5 μM CellTrace Violet (Invitrogen, Thermo Fisher Scientific) according to the manufacturer’s protocols and stimulated with human CD3^+^/CD28^+^ T cell activator (Stemcell Technologies) in the activation medium, XF T Cell Expansion Medium (Stemcell Technologies), for 40 hours. Upon completion of the 40-hour activation phase (defined as day 2), the activation medium was replaced with the proliferation medium, XF T Cell Expansion Medium plus 10 ng/mL IL-2 (Peprotech). Thereafter, the assessment of T cell activator–induced CD25/CD69 expression and T cell subpopulations was conducted via flow cytometric assays. Surface proteins (60) were stained with CD3-BV510 (clone 17A2, BioLegend); CD4-APC (clone SK3, BD Bioscience); CD8-BV605 (clone SK1, BioLegend); CD69-PerCP/Cyanine 5.5 (clone FN50, BioLegend); CD25-PE (clone 2A3, Stemcell Technologies); CD45RA-APC/Cy7 (clone HI100, BioLegend); and CCR7-PE/Cy7 (clone 2-L1-A, BD Biosciences). Concurrently, cell proliferation was determined by measuring the progressive dilution of CellTrace Violet (61) at specified cryopreservation durations on days 0 (before activation), 2 (the first day after activation), 3, 4, 5, 6, and 7.

### NK cell expansion and cytotoxicity.

NK cells expanded from UCB MNCs (removal of CD34^+^ cells using a CD34 microbead kit) were cultured in NK expansion medium, which was developed for the cultivation, activation, and expansion of human NK cells. Briefly, MNCs isolated from UCB were cultured in the NK expansion medium, which consisted of NK MACS Medium (Miltenyi Biotec); 5% human AB serum (MilliporeSigma); 500 U/mL IL-2 (Peprotech); and 140 U/mL IL-15 (Peprotech) for 21 days, while maintaining a cell density of 5 × 10^5^ cells/mL. To assess direct cytotoxicity, UCB-derived NK cells were incubated with target cells (K562 GFP reporter cell line, purchased from the Blood Biobank of Institute of Hematology and Blood Diseases Hospital) in U-bottomed, 96-well plates for 4 hours, utilizing effector-to-target (E/T) ratios 10:1, 5:1, 2.5:1, 0.625:1, and 0:1. Subsequently, the cells were suspended in PBS for flow cytometric analysis. The ratio of CD3^–^CD56^+^ cells to GFP^+^ cells before and after coculturing was indicative of the residual proportion of target cells.

### IL-2, TNF-α, GZMB, and perforin staining.

To induce cytokine production, magnetically enriched CD3^+^ T cells or UCB expanded NK cells were incubated with or without PMA and ionomycin (MilliporeSigma) for 4 hours (34). Following this stimulation, the cells were subjected to staining for surface markers, and subsequent fixation was carried out using fixation/permeabilization concentrate (eBioscience) according to the manufacturer’s instructions. Thereafter, intracellular staining was performed to assess the presence of IL-2 (PerCP/Cyanine 5.5, clone MQ1-17H12, BioLegend), TNF-α (PE, clone mAb11, BioLegend), granzyme B (GZMB) (FITC, clone QA16A02, BioLegend), and perforin (APC, clone B-D48, BioLegend).

### Cell culturing and sulforaphane treatment.

For sulforaphane treatment in vitro, UCB CD34^+^ cells were cultured with StemSpan Serum-Free Expansion Medium (SFEM) (Stemcell Technologies), 50 ng/mL TPO, 100 ng/mL SCF, 50 ng/mL FLT-3, and cultured with or without 2.5 μM SF for approximately 40 hours. After this treatment, a colony-forming assay, liquid culturing, and xenotransplantation procedures were conducted.

### Cellular translation rate analysis.

CD34^+^ cells isolated from UCB were seeded into 48-well plates to study blood cell metabolism. The cells were plated at a concentration of 1 × 10^6^ cells/mL in 300 μL DMEM medium per well. Puromycin (puro) (final concentration 10 μg/mL) was added to the medium and incubated for 40 minutes. Cells were then washed twice with cold PBS. Surface marker staining was performed using CD34-FITC (clone 581, BD Biosciences) and CD38-PE/Cy7 (clone HB7, BD Biosciences). After washing, cells were fixed and permeabilized using FOXP3 fixation and permeabilization buffer (Thermo Fisher Scientific) according to the manufacturer’s instructions. Intracellular staining for puro was performed using Alexa Fluor 647 anti-puro monoclonal antibody (clone 2A4, BioLegend) (62). Cells were incubated with the antibody for 1 hour at 4°C and then diluted 1:100 in permeabilization buffer.

### UCB cryopreservation and sulforaphane treatment.

A unit of cord blood was partitioned into 2 equitably sized portions. One group was subjected to cryopreservation with 10 μM sulforaphane (S4441, MilliporeSigma) incorporated into the freezing solution (SF group), whereas the control group underwent cryopreservation without the addition of SF, and both groups underwent the following cryopreservation procedures: hydroxyethyl starch at a volume of 1:5 was introduced to UCB for RBC removal, allowing sedimentation at room temperature for 40 minutes. Subsequently, the supernatant was aspirated, and the cells were centrifuged, with the majority of the supernatant discarded to achieve a total volume of 32 mL. Following this, 8 mL CryoSure-DEX40 (WAK-Chemie Medical) cryopreservation solution, with SF or DMSO, was rapidly introduced, ensuring thorough mixing before the solution was transferred into a cryopreservation bag. Using the CryoMed Controlled-Rate Freezers (Thermo Fisher Scientific), the temperature of the UCB was systematically reduced, culminating in its ultimate storage in liquid nitrogen.

### Statistics and plots.

Nonparametric Wilcoxon tests were used to measure the difference between 2 groups with R language. The reported *P* values were determined by 2-sided tests, and a *P* value of less than 0.05 was considered significant. UMAP plots were generated using Scanpy or Seurat. Line plots, bubble plots, bar plots, box plots, dot plots, etc., were generated in the ggplot2 package, whereas radar plots were implemented in the ggradar package. GraphPad Prism 9.5 (GraphPad Software) was applied to perform experimental statistical analyses and plots. For experiments comparing 2 groups, statistical differences were assessed using an unpaired, 2-tailed *t* test or the Wilcoxon test. For experiments involving 3 or more groups, data were analyzed with a 1-way ANOVA or a Kruskal-Wallis test followed by a post hoc test based on the data distribution for multiple comparisons. Data from experiments are presented as the mean ± SEM, and bioinformatics analyses are presented as the mean ± SD.

### Study approval.

All human studies were approved by the Institute of Hematology and Blood Diseases Hospital, Chinese Academy of Medical Sciences and Peking Union Medical College, Tianjin, China. UCB sample collection and processing and laboratory operations were approved by the ethics committee of the Institute of Hematology and Blood Disease Hospital. All animal experiments were conducted in accordance with protocols approved by the Institute of Hematology Animal Care and Use Committee, Tianjin, China.

### Data availability.

The single-cell data in this study have been deposited in the National Genomics Data Center, Genome Sequence Archive (GSA) (accession code HRA006642; https://ngdc.cncb.ac.cn/gsa-human/s/UV0No2h8). Supporting data relevant to the main manuscript and supplemental materials are available in the Supporting Data Values file.

## Author contributions

TC, Yingchi Zhang, and FD supervised the project. Y Huang performed the experiments with the help of Yawen Zhang and J Ye. XX and ML performed the bioinformatics analysis. J Yang, WY, GL, and SM provided the cryopreserved UCB. ZS and XZ contributed the clinical data and JC, Y Hu, SQ, and YF collected and conducted statistical analyses of the clinical samples. HC, LH, SL, Lin Wang, Lu Wang, JS, and XP contributed to the study through discussions that assisted in the development of the research concept. TC, Yingchi Zhang, FD, XX, and Y Huang wrote the manuscript. All authors discussed the results and commented on the manuscript. The order of the co–first authors was determined based on their equal contributions to the research.

## Supplementary Material

Supplemental data

Supplemental data set 1

Supplemental data set 2

Supporting data values

## Version 1 03/04/2025

In-Press Preview

## Version 2 05/01/2025

Electronic publication
