# Supplementary material for: Restoring mitochondrial function promotes hematopoietic reconstitution from cord blood following cryopreservation-related functional decline
Source: J Clin Invest. 2025 Mar 4;135(9):e183607. doi: 10.1172/JCI183607 (PMC12043090; doi:10.1172/JCI183607)

Supplemental Figure 1. Single-cell transcriptomic profiles of HSPCs and MNCs from fresh and cryopreserved UCB

(A) Cell viability of $CD34^+$ cells and mononuclear cells from fresh and cryopreserved UCB stored for 1, 5, 10, and 19 years before scRNA-seq. **(B)** UMAP plots displaying distributions of single cells from fresh and cryopreserved UCB stored for 1, 5, 10, and 19 years. **(C)** UMAP plots displaying distributions of single cells from HSPC and MNC. **(D-G)** UMAP plots displaying distributions of single cells from different storage years for (D) HSPCs, (E) T/NK cells, (F) B cells and (G) monocytes.

Supplemental Figure 2. Single-cell transcriptomic profiles of HSPCs and MNCs from fresh and cryopreserved UCB

(A) Bubble plot showing expression levels of signature genes in each cluster of HSPCs. **(B-C)** Cell distributions (B) and signature gene expressions (C) of 8 cell clusters of T/NK cells. **(D-E)** Cell distributions (D) and signature gene expressions (E) of 6 cell clusters of B cells. **(F-G)** Cell distributions (F) and signature gene expressions (G) of 8 cell clusters of monocytes. **(H)** Cellular abundances of 8 cell clusters of T/NK cells from fresh and cryopreserved UCB stored for 1, 5, 10, and 19 years (n = 4 replicates; error bars indicate mean ± SD). **(I)** Cellular abundances of 6 cell clusters of B cells from fresh and cryopreserved UCB stored for 1, 5, 10, and 19 years (n = 4 replicates; error bars indicate mean ± SD). **(J)** Cellular abundances of 8 cell clusters of monocytes from fresh and cryopreserved UCB stored for 1, 5, 10, and 19 years (n = 4 replicates; error bars indicate mean ± SD). **(K)** Cell distribution and cellular abundances of 9 cell

clusters of HSPCs from fresh and cryopreserved UCB collected from Shandong Qilu Stem Cell Engineering Co., Ltd. (n = 4 replicates; error bars indicate mean ± SD). **(L)** Representative flow cytometry plots and frequency of early apoptotic cells (Annexin^{+} PI^{-}) and late apoptotic cells (Annexin^{+} PI^{+}) in CD34^{+} population from fresh and cryopreserved UCB (Y10) (n = 2 replicates; error bars indicate mean ± SEM; two-tailed t test).

Supplemental Figure 3. Transcriptomic variations occurred in UCB during cryopreservation

(A) Differences in number of DEGs between any two cryopreservation periods for HSPCs, T/NK cells, B cells, and monocytes. The dotted line represents the median of DEGs in the Mito^{hi} subpopulations. **(B)** Differences in number of DEGs between two consecutive cryopreservation periods for T/NK cells (top), B cells (middle), and monocytes (bottom). **(C)** Bubble plot showing enriched GO terms of upregulated genes in HSPCs from fresh UCB (fresh UCB versus cryopreserved UCB, with UCB samples of different cryopreservation times regarded as a whole). **(D)** Differentiation potential towards MLP, GMP, and MEP lineages of HSCs from fresh and cryopreserved UCB, with UCB samples of different cryopreservation times regarded as a whole. **(E)** The number of DEGs between the HSPCs of fresh and cryopreserved UCB from Shandong Qilu Stem Cell Engineering Co., Ltd.. **(F)** Enriched GO terms of upregulated genes in Mito^{hi} HSC/MPPs from fresh UCB (left) and cryopreserved UCB (right) from Shandong Qilu Stem Cell Engineering Co., Ltd.. **(G)** Percentages of HSC, MPP1,

MPP2, and Mito^{hi} HSC/MPP cells in different cell- cycle phases from fresh and cryopreserved UCB from Shandong Qilu Stem Cell Engineering Co., Ltd. (n = 4 replicates; error bars indicate mean ± SD). For each cluster, the proportions of cells in G2M phase at Y10 was compared with that of fresh UCB using the Chi-squared test (Supporting data values). **(H)** Relative expression of stemness signature genes in HSC, MPP1, MPP2, and Mito^{hi} HSC/MPP cells from fresh and cryopreserved UCB from Shandong Qilu Stem Cell Engineering Co., Ltd.. The stemness signature genes used for the analysis were provided in Data file S2. **(I)** Differentiation potential towards MLP, GMP and MEP lineages of HSCs (left) and Mito^{hi} HSC/MPPs (right) from fresh and cryopreserved UCB from Shandong Qilu Stem Cell Engineering Co., Ltd.

Supplemental Figure 4. Decreased functions of HSPCs after cryopreservation

(A) CFCs per 200 CD34^{+} cells from fresh and cryopreserved UCB stored for different years with individual UCB (Fresh: 8 replicates; Y1 and Y5: 5 replicates; Y10: 12 replicates; Y19: 6 replicates). **(B)** Representative flow cytometry plots of human cell engraftment in BM of recipients 20 weeks after transplantation. **(C-D)** Percentage of human CD45^{+} cell engrafted in spleen (SP) of (C) primary and (D) secondary recipients. **(E-F)** Lineage differentiation potential of human hematopoietic cells in (E) BM and (F) SP of primary (top) and secondary (bottom) recipients. B: hCD45^{+}CD19^{+} B cells, M: hCD45^{+}CD33^{+} myeloid cells, T: hCD45^{+}CD3^{+} T cells. NK: hCD45^{+}CD56^{+} NK cells. **(G)** CFCs per 200 CD34^{+} cells from fresh and cryopreserved UCB (3 weeks) (5 replicates). **(H)** CFCs per 200 CD34^{+} cells from fresh and cryopreserved UCB (Y10)

from Shandong Qilu Stem Cell Engineering Co., Ltd. Data from four independent UCB samples (4 replicates). **(I)** Flow chart of patient selection. **(J)** Proportion of engrafted NCG-X mice with successful platelet reconstruction. Successful engraftment was defined as > 0.1% human $CD41a^+$ platelets in recipient PB (4 pooled UCB samples, 4-7 recipient mice per group). **(K)** Number of human platelets in PB (10^3 platelets/μl) of recipients after 10 weeks transplantation. **(L)** Burst-forming unit-erythroid (BFU-E) per 200 $CD34^+$ cells from fresh and cryopreserved UCB (Fresh: 8 replicates; Y1: 5 replicates; Y5: 4 replicates; Y10: 12 replicates; Y19: 6 replicates). **(M)** Percentage of human $CD235a^+$ $CD71a^+$ cells engrafted in the BM of recipients (NCG-X mice). 4 pooled UCB samples; 6-9 recipient mice per group. For transplantation assay, UCB derived $CD34^+$ cells (40,000 /mouse) were injected via the tail vein.

(C-F) 4-6 pooled UCB samples; 15-24 recipient mice per group. **(C-H, K)** Error bars indicate mean ± SEM; two-tailed t test. *, $p \leq 0.05$; **, $p \leq 0.01$; ***, $p \leq 0.001$.

Supplemental Figure 5. T and NK cells derived from UCB were resistant to cryopreservation

(A) Relative expression levels of canonical naïve- and cytotoxicity-associated genes (*TCF7, CD27,* and *KLRD1*) in fresh and cryopreserved NK cells and T cells. Wilcoxon test. **, $p \leq 0.01$; ****, $p \leq 0.0001$. **(B)** CD69 expression in $CD3^+$ T cells, with or without human CD3/CD28 activator, from fresh and cryopreserved UCB stored for 10 years (n = 6 replicates, three independent experiments). **(C)** Histograms showing cell division count of $CD3^+$ T cells of fresh and cryopreserved UCB stored for 10 years over

the 8 days (d2-d7) following CD3/CD28 stimulation (n = 2 replicates, three independent experiments). **(D)** Flow cytometry plots depicting distinct subpopulations of $CD3^{+}CD8^{+}$ T cells at the 40-hour time point following CD3/CD28 activation. **(E)** Frequency of subpopulations of $CD3^{+}CD8^{+}$ T cells (n = 2 replicates, three independent experiments). **(F)** Expression of GZMB and perforin in expanded $CD3^{-}CD56^{+}$ cells, with or without PMA and ionomycin treatment, from fresh and cryopreserved UCB stored for 10 years (fresh, n = 4 replicates; Y10, n = 4 replicates. 3 independent experiments).

(B-C, E-F) Error bars indicate mean ± SEM; two-tailed t test. *, $p \leq 0.05$; **, $p \leq 0.01$; ***, $p \leq 0.001$; ****, $p \leq 0.0001$.

Supplemental Figure 6. Abnormal mitochondrial metabolism in cryopreserved HSPCs

(A) Relative expression of oxidative phosphorylation genes, mitochondrial respiratory chain complex assembly and reactive oxygen species biosynthetic process genes in $Mito^{hi}$ $CD4^{+}$ naive T cells from fresh and cryopreserved UCB. For each cluster: Y1, Y5, Y10, and Y19 samples were respectively compared with the fresh UCB using the Kruskal-Wallis test. **(B)** Representative flow cytometry plots of mitochondrial membrane potential of HSC/MPPs cells from fresh and cryopreserved UCB (Y10), as determined with TMRE staining. **(C)** Relative expression of mitochondria-related genes in fresh and cryopreserved (Y10) HSC/MPPs with low and high TMRE signals. Wilcoxon test. **(D)** Bubble plot showing upregulated gene sets in the HSC/MPP clusters

with low and high TMRE signals. NESs were obtained by GSEA analysis. **(E)** Representative flow cytometry plots of mitochondrial ROS levels of $CD34^+$ cells from fresh and cryopreserved UCB (Y5 and Y10). **(F)** Percentage of $MitoSox^+$ in $CD34^+$ cells from fresh and cryopreserved UCB stored for 3 weeks (2-3 replicates, two independent experiments). **(G)** ECR of $CD34^+$ cells from fresh and cryopreserved UCB (including Y5 and Y10; fresh, 3 pooled UCB, 6 technical replicates; Y5, 4 pooled UCB, 2 technical replicates ; Y10, 4 pooled UCB, 2 technical replicates). **(H)** Mitochondrial morphology, as analyzed by MiNA, of $CD34^+$ cells from fresh and cryopreserved UCB stored for 10 years: mean branches per network (left) and number of individuals (right). Fresh: 90 cells from 3 pooled UCB; Y10: 104 cells from 3 pooled UCB. **(I)** OCR of $CD3^+$ cells, induced by CD3/CD28 stimulation, from fresh and cryopreserved UCB (Y5 and Y10) (fresh, 3 pooled UCB, 4 technical replicates; Y10, 3 pooled UCB, 2 technical replicates).

(F-I) Error bars indicate mean ± SEM; two-tailed t test. *, $p \leq 0.05$; **, $p \leq 0.01$; ****, $p \leq 0.0001$.

Supplemental Figure 7. Sulforaphane improved the function of cryopreserved UCB-derived HSPCs

(A) CFCs per 200 $CD34^+$ cells from cryopreserved UCB stored for 10 years cultured with different antioxidants for 40 hours, 4 replicants. Antioxidant concentration, Ctrl: same volume of DMSO; Nac: 0.1 mM; Res: 0.2 μM; SF: 2.5 μM; VitE: 10 mg/ml, 2 mg/ml, 1 mg/ml; CoQ10:10 nM, 5 nM, 1 nM. The red dot plot represents the mean

CFCs of the control group. **(B)** Representative flow cytometry plots of $hCD45^+$ and megakaryocyte engrafted in BM of recipients 12 weeks post-transplantation from UCB $CD34^+$ cells cryopreserved for 10 years (Y10), cultured with (right) or without (left) SF. **(C)** Percentage of human $CD45^+$ cells from UCB cryopreserved for fresh and 10 years (Y10) engrafted in spleen of mouse recipients at 12 weeks after transplantation in SF-untreated and SF-treated groups (2-4 pooled UCB samples; 6-10 recipient mice per group). **(D-E)** Lineage differentiation potential of human hematopoietic cells in (D) BM and (E) SP of recipients in SF-untreated and SF-treated group (B: $hCD45^+CD19^+$ B cells, M: $hCD45^+CD33^+$ myeloid cells, T: $hCD45^+CD3^+$ T cells. 2-4 pooled UCB samples; 6-10 recipient mice per group). **(F)** Number of human platelets in PB (10^2 platelets/μl) of recipients after 12 weeks transplantation. **(G)** Representative image of colony-forming cells of control and SF groups captured by Nikon Ti-E Living Cell Workstation. **(H)** Representative flow cytometry plots of mitochondrial ROS levels of $CD34^+$ cells from blank (MitoSox unstained), control and SF group (left). Percentage of $MitoSox^+$ cells in $CD34^+$ population from control and SF groups (right; 2 biological replicates).

(C-F) Error bars indicate mean ± SEM; two-tailed t test. *, $p \leq 0.05$.

151 **Supplemental Table 1. Characteristics of the study cohort, related to Figure 3H.**

152 Subject co-variates (n = 171)

Year at transplant, n (%)	
2015	41 (24)
2016	22 (13)
2017	22 (13)
2018	26 (15)
2019	28 (16)
2020	32 (19)
Age, years, median (range)	9.6 (0.7–55.4)
Male, n (%)	105 (61)
Leukemia state, n (%)	
CR1	95 (56)
Not CR1	76 (44)
HLA mismatch in GvHD direction, n (%)	
None	8 (5)
1 locus	25 (15)
2 loci	48 (28)
3 loci	48 (28)
4 loci	31 (18)
5 loci	10 (6)
6 loci	1 (1)

CD34-positive/BW cell dose, 10E+5 cells/kg, median (range)	4.2 (3.0 – 10.9)
Cryopreservation duration, years, median (range)	2.6 (0.8 – 13.6)

Abbreviations: BW, body weight; CR, complete remission; GvHD, graft-versus-host disease.

Data file S1. Signature genes of HSPCs, T/NK cells, B cells, and monocytes, related to Figure 1 and Supplemental Figure 1.

Data file S2. Genes included in stemness signature analysis, related to Figure 2D and Supplemental Figure 3H.

Supplemental Figure 1

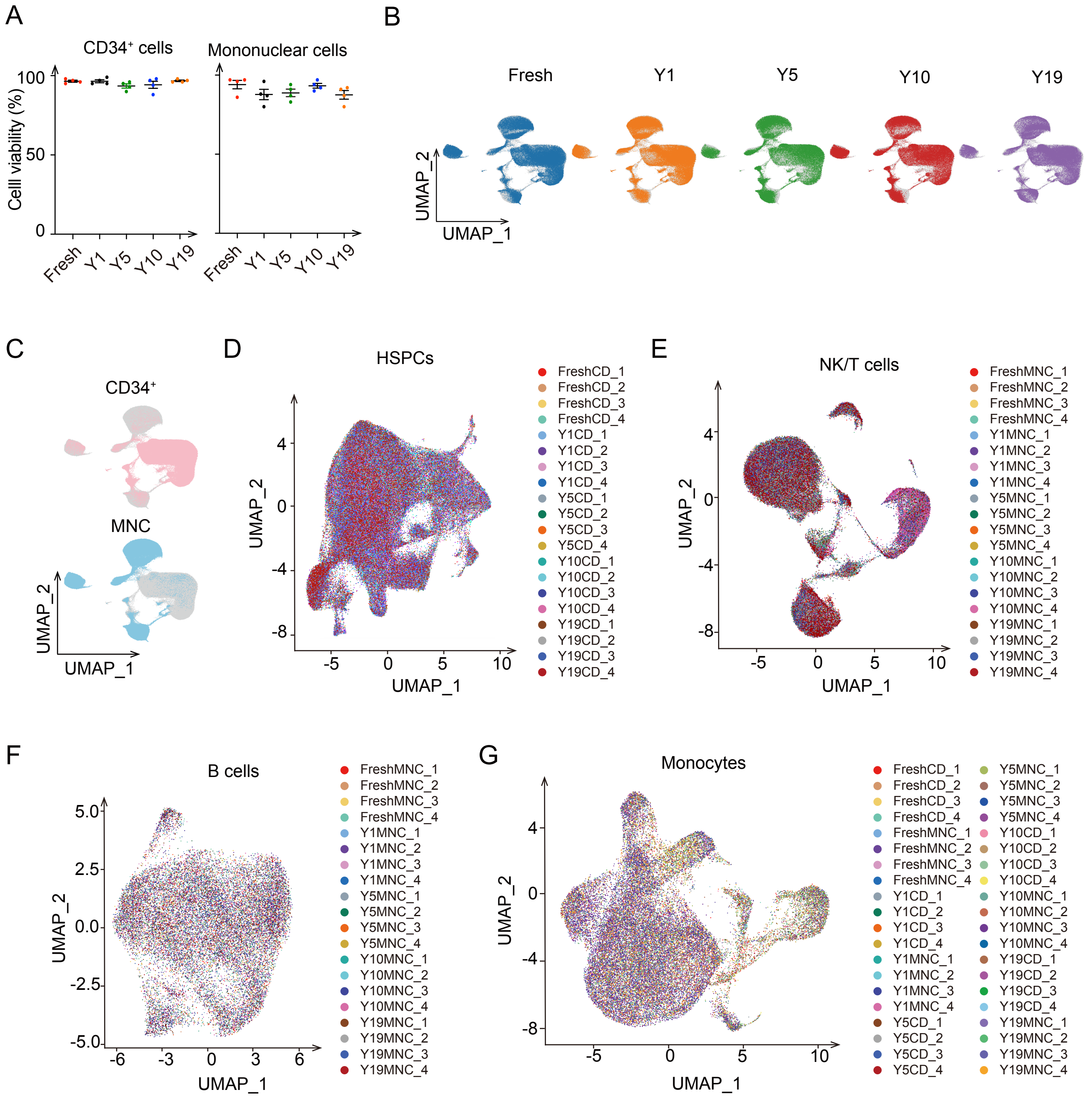

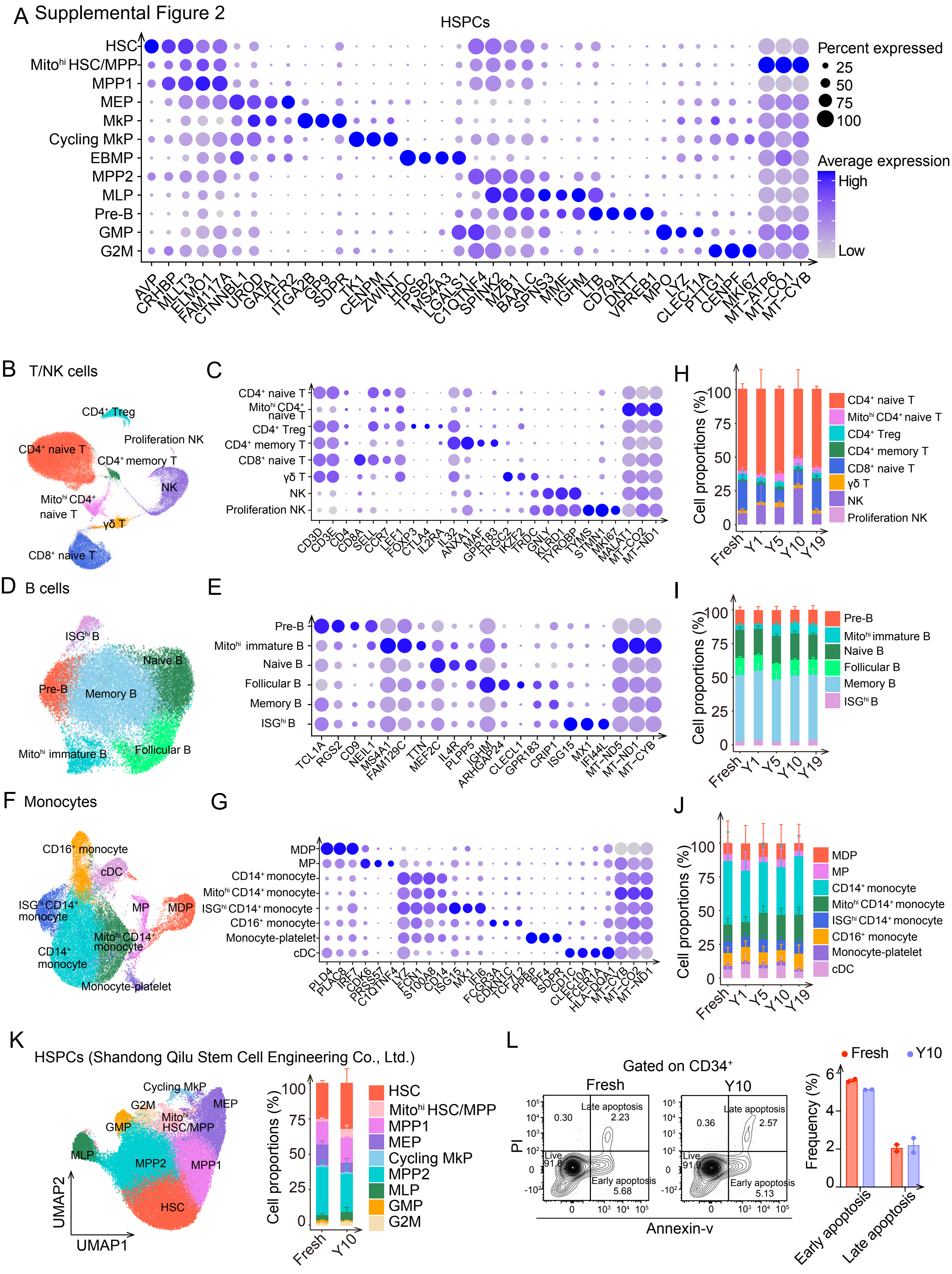

A Supplemental Figure 2
HSPCs
HSC
Mitohi HSC/MPP
MPP1
MEP
MkP
Cycling MkP
EBMP
MPP2
MLP
Pre-B
GMP
G2M
Percent expressed
25
50
75
100
Average expression
High
Low
AVP
CRHBP
MLLT3
ELMO1
FAM117A
CTNNBL1
UROD
GATA1
TFR2
ITGA2B
GP9
SDPR
TK1
CENPM
ZWINT
HDC
TPSB2
MS4A3
LGALS1
C1QTNF4
SPINK2
MZB1
BAALC
SPNS3
MME
IGHM
LTB
CD79A
DNTT
VPREB1
MPO
LYZ
CLEC11A
PTTG1
CENPF
MKI67
MT-ATP6
MT-CO1
MT-CYB
B T/NK cells
CD4+ Treg
Proliferation NK
CD4+ naive T
CD4+ memory T
NK
Mitohi CD4+ naive T
γδ T
CD8+ naive T
C
Mitohi CD4+ naive T
CD4+ Treg
CD4+ memory T
CD8+ naive T
γδ T
NK
Proliferation NK
CD3D
CD3E
CD4
CD8A
SELL
CCR7
LEF1
FOXP3
CTLA4
IL2RA
IL32
ANXA1
MAF
GPR183
TRGC2
IKZF2
TRDC
GNLY
KLRD1
TYROBP
TYMS
STMN1
MKI67
MALAT1
MT-CO2
MT-ND1
H
Cell proportions (%)
100
75
50
25
0
Fresh
Y1
Y5
Y10
Y19
D B cells
ISGhi B
Naive B
Pre-B
Memory B
Mitohi immature B
Follicular B
E
Pre-B
Mitohi immature B
Naive B
Follicular B
Memory B
ISGhi B
TCL1A
RGS2
CD9
NEIL1
MS4A1
FAM129C
TTN
MEF2C
IL4R
PLPP5
IGHM
ARHGAP24
CLECL1
GPR183
CRIP1
ISG15
MX1
IFI44L
MT-ND5
MT-ND1
MT-CYB
I
F Monocytes
CD16+ monocyte
cDC
ISGhi CD14+ monocyte
MP
MDP
Mitohi CD14+ monocyte
CD14+ monocyte
Monocyte-platelet
G
MDP
MP
CD14+ monocyte
Mitohi CD14+ monocyte
ISGhi CD14+ monocyte
CD16+ monocyte
Monocyte-platelet
cDC
PLD4
PLAC8
IRF7
CDK6
PRSS57
C1QTNF4
LYZ
FCN1
S100A8
CD14
ISG15
MX1
IFI6
FCGR3A
CDKN1C
TCF7L2
PPBP
PF4
SDPR
CD1C
CLEC10A
FCER1A
HLA-DQA1
MT-CYB
MT-CO2
MT-ND1
J
K
HSPCs (Shandong Qilu Stem Cell Engineering Co., Ltd.)
Cycling MkP
G2M
Mitohi HSC/MPP
MEP
GMP
MLP
MPP2
MPP1
HSC
UMAP2
UMAP1
HSC
Mitohi HSC/MPP
MPP1
MEP
Cycling MkP
MPP2
MLP
GMP
G2M
L
Gated on CD34+
Fresh
Y10
0.30
Late apoptosis
2.23
0.36
2.57
Live
91.8
91.9
Early apoptosis
5.68
5.13
PI
Annexin-v
Frequency (%)
Early apoptosis
Late apoptosis

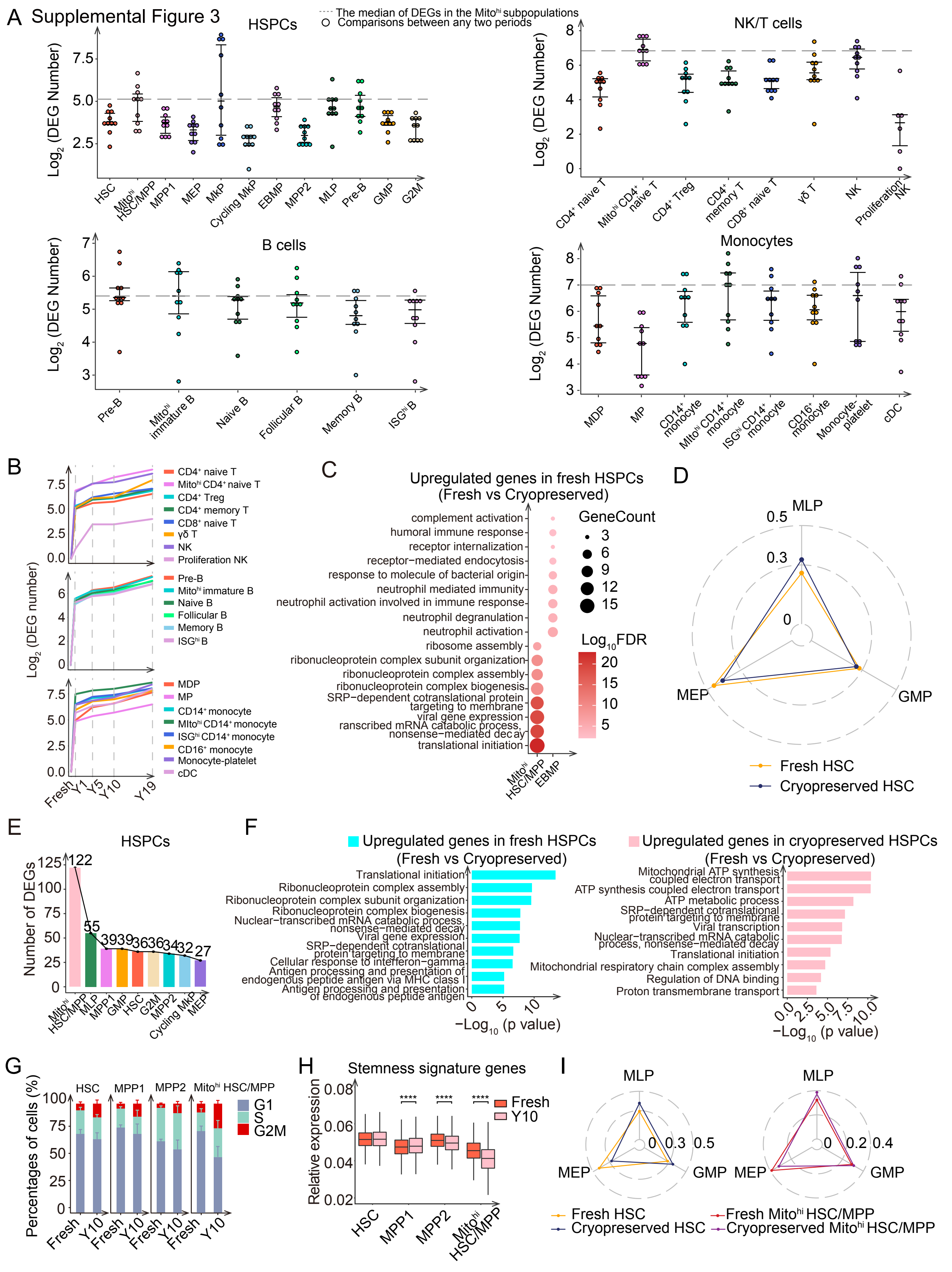

A
Supplemental Figure 3
HSPCs
The median of DEGs in the Mito^hi subpopulations
Comparisons between any two periods
Log2 (DEG Number)
HSC
Mito^hi HSC/MPP
MPP1
MEP
MkP
Cycling MkP
EBMP
MPP2
MLP
Pre-B
GMP
G2M
NK/T cells
CD4+ naive T
Mito^hi CD4+ naive T
CD4+ Treg
CD4+ memory T
CD8+ naive T
γδ T
NK
Proliferation NK
B cells
Pre-B
Mito^hi immature B
Naive B
Follicular B
Memory B
ISG^hi B
Monocytes
MDP
MP
CD14+ monocyte
Mito^hi CD14+ monocyte
ISG^hi CD14+ monocyte
CD16+ monocyte
Monocyte-platelet
cDC
B
Log2 (DEG number)
CD4+ naive T
Mito^hi CD4+ naive T
CD4+ Treg
CD4+ memory T
CD8+ naive T
γδ T
NK
Proliferation NK
Pre-B
Mito^hi immature B
Naive B
Follicular B
Memory B
ISG^hi B
MDP
MP
CD14+ monocyte
Mito^hi CD14+ monocyte
ISG^hi CD14+ monocyte
CD16+ monocyte
Monocyte-platelet
cDC
Fresh
Y1
Y5
Y10
Y19
C
Upregulated genes in fresh HSPCs
(Fresh vs Cryopreserved)
complement activation
humoral immune response
receptor internalization
receptor−mediated endocytosis
response to molecule of bacterial origin
neutrophil mediated immunity
neutrophil activation involved in immune response
neutrophil degranulation
neutrophil activation
ribosome assembly
ribonucleoprotein complex subunit organization
ribonucleoprotein complex assembly
ribonucleoprotein complex biogenesis
SRP−dependent cotranslational protein targeting to membrane
viral gene expression
ranscribed mRNA catabolic process, nonsense−mediated decay
translational initiation
Mito^hi HSC/MPP
EBMP
GeneCount
Log10FDR
D
MLP
MEP
GMP
Fresh HSC
Cryopreserved HSC
E
HSPCs
Number of DEGs
122
55
39
39
36
36
34
32
27
Mito^hi HSC/MPP
MLP
MPP1
GMP
HSC
G2M
MPP2
Cycling MkP
MEP
F
Upregulated genes in fresh HSPCs
(Fresh vs Cryopreserved)
Translational initiation
Ribonucleoprotein complex assembly
Ribonucleoprotein complex subunit organization
Ribonucleoprotein complex biogenesis
Nuclear−transcribed mRNA catabolic process, nonsense−mediated decay
Viral gene expression
SRP−dependent cotranslational protein targeting to membrane
Cellular response to interferon−gamma
Antigen processing and presentation of endogenous peptide antigen via MHC class I
Antigen processing and presentation of endogenous peptide antigen
−Log10 (p value)
Upregulated genes in cryopreserved HSPCs
(Fresh vs Cryopreserved)
Mitochondrial ATP synthesis coupled electron transport
ATP synthesis coupled electron transport
ATP metabolic process
SRP−dependent cotranslational protein targeting to membrane
Viral transcription
Nuclear−transcribed mRNA catabolic process, nonsense−mediated decay
Translational initiation
Mitochondrial respiratory chain complex assembly
Regulation of DNA binding
Proton transmembrane transport
−Log10 (p value)
G
HSC
MPP1
MPP2
Mito^hi HSC/MPP
Percentages of cells (%)
G1
S
G2M
Fresh
Y10
H
Stemness signature genes
Relative expression
Fresh
Y10
****
HSC
MPP1
MPP2
Mito^hi HSC/MPP
I
MLP
MEP
GMP
Fresh HSC
Cryopreserved HSC
Fresh Mito^hi HSC/MPP
Cryopreserved Mito^hi HSC/MPP

Supplemental Figure 4

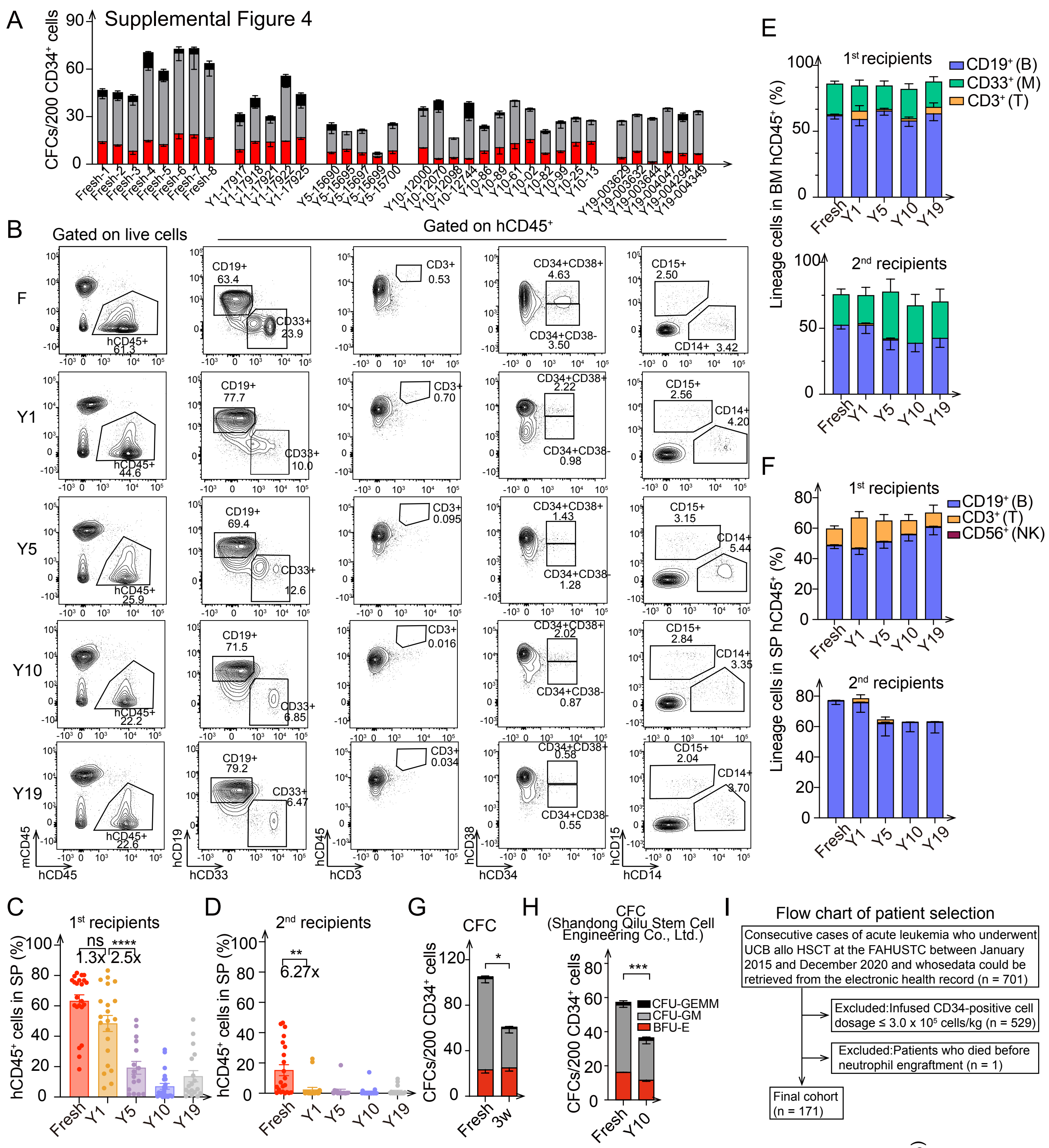

J

Time points (after transplantation)	Fresh (human platelet engraftment in PB)	Y10 (human platelet engraftment in PB)
1 week	0/4	0/8
3 weeks	4/4	3/7
5 weeks	4/4	5/7
7 weeks	4/4	7/7
9 weeks	4/4	7/7

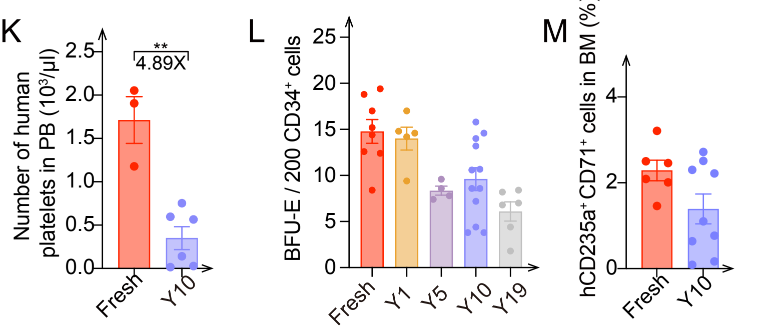

Supplemental Figure 5

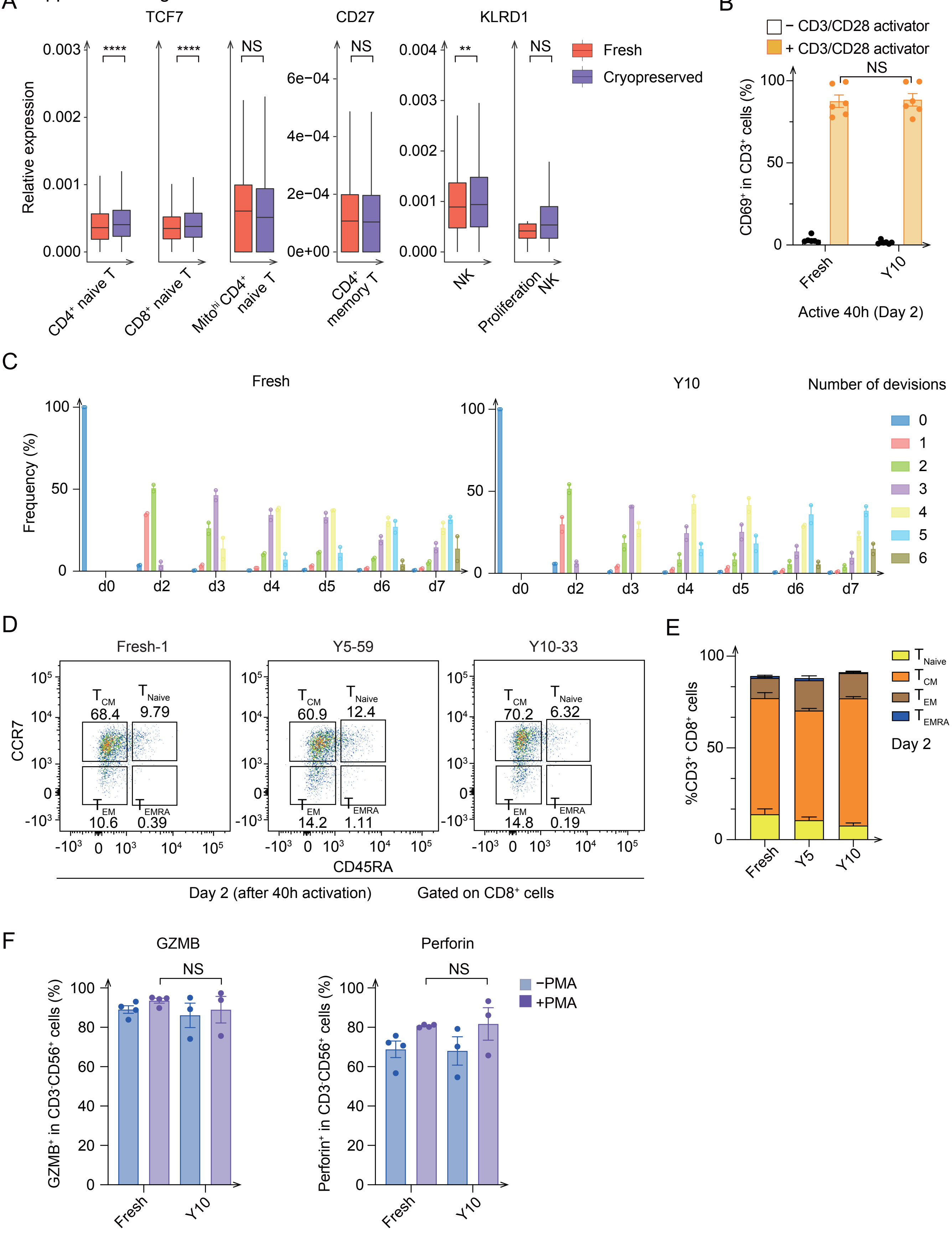

Supplemental Figure 6

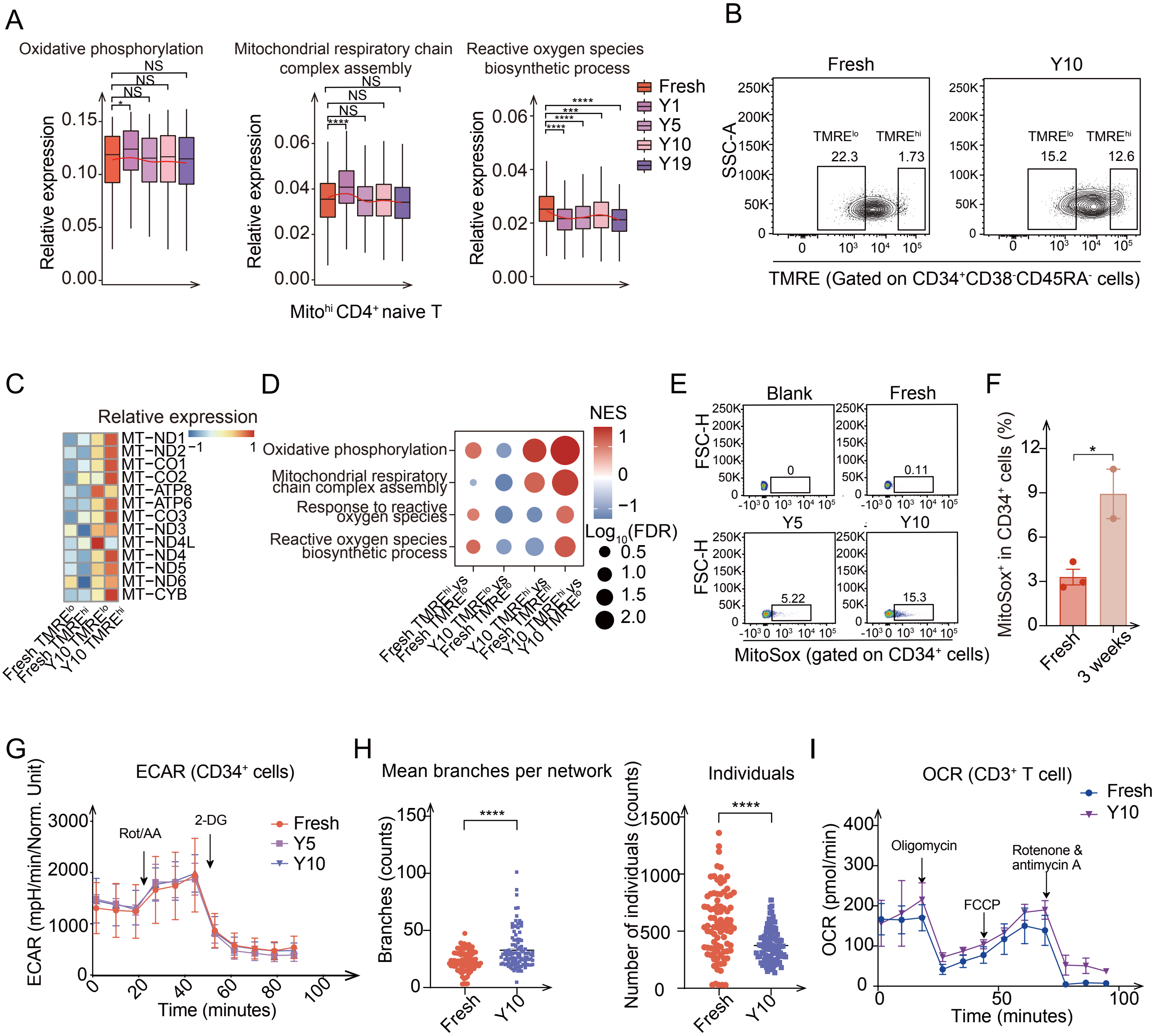

Supplemental Figure 7

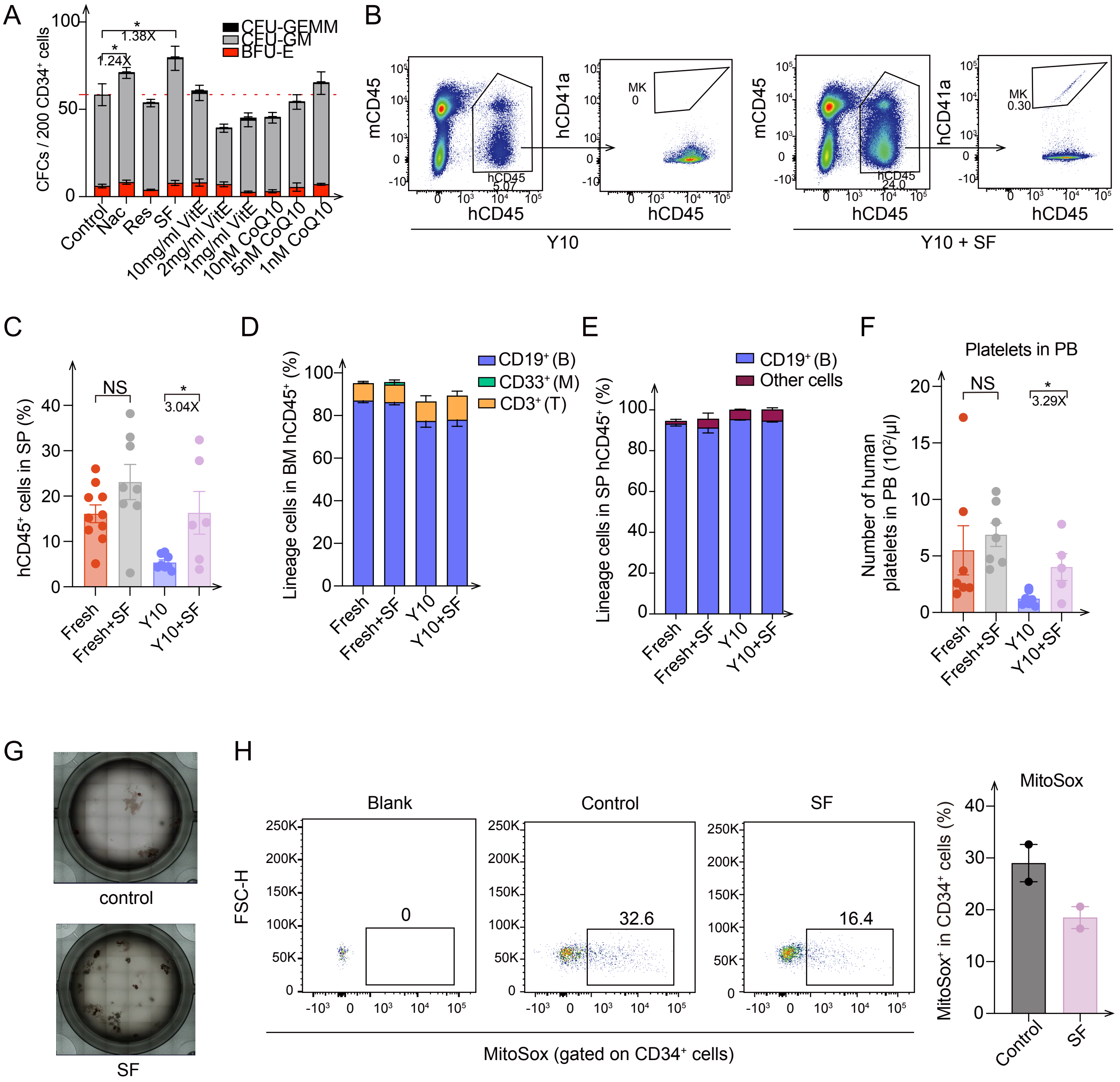

Supplement: Supplemental data [file jci-135-183607-s019.pdf]
