# Supplementary material for: Restoring mitochondrial function promotes hematopoietic reconstitution from cord blood following cryopreservation-related functional decline
Source: J Clin Invest. 2025 Mar 4;135(9):e183607. doi: 10.1172/JCI183607 (PMC12043090; doi:10.1172/JCI183607)
Supplement: Supplemental data set 2 [file jci-135-183607-s021.pdf]

ENO1
CD52
PHACTR4
MARCKSL1
CSF3R
PRDX1
JUN
NFIA
LEPROT
GADD45A
PRKACB
GBP4
S100A10
CD48
PBX1
SELL
PTPRC
MIR181A1HG
CD34
LAPTM4A
ZFP36L2
SPTBN1
VAMP8
GYPC
ZEB2
TFPI
SLC40A1
IKZF2
ITM2C
ANKRD28
MAPKAPK3
TKT
NFKBIZ
ZBTB20
GATA2
CPA3
HOPX
SPINK2
IGFBP7
EREG
AREG
HSD17B11
HPGDS
ANXA5
LRBA
NPR3
CRHBP
EPB41L4A−AS1
H2AFY
MZB1

DUSP1
RNF130
SERPINB1
HLA-E
HLA-DRA
HLA-DRB5
HLA-DRB1
HLA-DQA1
HLA-DQB1
HLA-DPA1
HLA-DPB1
CD109
PNRC1
ADGRG6
MYCT1
ELMO1
LAT2
HSPB1
CDK6
AC002454.1
GNG11
FAM3C
TRBC2
CD99
TMSB4X
ITM2A
BEX1
LAPTM4B
BAALC
EIF3E
MLLT3
ANXA1
SNHG7
NPDC1
IFITM3
LSP1
NUCB2
LDHA
MDK
C1QTNF4
FTH1
GSTP1
SESN3
ZBTB16
CELF2
VIM
MAP3K8
SRGN
PDZD8
GAPDH

CD69
NR4A1
TESPA1
MSRB3
HMGA2
SOCS2
ATP6V0A2
POMP
TPT1
COMMD6
TNFSF13B
PSME1
PSME2
MEG3
CKB
PKM
CPPED1
CORO1A
CYBA
SPNS3
FAM117A
HLF
DGKE
ACTG1
AVP
SNX5
ADA
ZMYND8
ZFAS1
CEBPB
SMIM24
PRAM1
ICAM3
KLF2
BST2
HCST
IGLL1
LGALS1
NRIP1
